# Tunable Interference Colors in Nanofibril–Crystal Composite Films via Integrated Salt-Assisted Assembly

**DOI:** 10.34133/research.1198

**Published:** 2026-03-20

**Authors:** Shaohuang Chen, Qun Song, Zengbin Wang, Yinqiang Xia, Philip Biehl, Rongxin Su, Kai Zhang

**Affiliations:** ^1^ Sustainable Materials and Chemistry, Department of Wood Technology and Wood-based Composites, University of Göttingen, 37077 Göttingen, Germany.; ^2^ State Key Laboratory of Chemical Engineering and Low-Carbon Technology, Tianjin Key Laboratory of Membrane Science and Desalination Technology, School of Chemical Engineering and Technology, Tianjin University, Tianjin 300072, China.; ^3^ College of Food Science and Engineering, Northwest A&F University, Yangling, Shaanxi 712100, China.; ^4^ Ningbo Key Laboratory of Green Petrochemical Carbon Emission Reduction Technology and Equipment, Zhejiang Institute of Tianjin University, Ningbo 315201, China.; ^5^ Tianjin Key Laboratory for Marine Environmental Research and Service, School of Marine Science and Technology, Tianjin University, Tianjin 300072, China.; ^6^ Wöhler Research Institute for Sustainable Chemistry (WISCh), Faculty of Chemistry, University of Göttingen, 37077 Göttingen, Germany.

## Abstract

Cellulose nanofibrils (CNFs) are strong, flexible biopolymers, whereas producing large-area (centimeter-scale) optical films with tunable, vivid interference colors with them remains difficult. The amorphous regions within the fibrils reduce birefringence and prevent uniform color formation. Here, we develop an integrated salt-assisted assembly (iSAA) strategy that combines metal-ion crosslinking with organic–inorganic coassembly to fabricate nanofibril–crystal composite films with tunable, vibrant colors. By adjusting the concentration of aluminum salt during assembly, we precisely control optical phase retardation from about 400 nm to over 2,500 nm, covering the full range of the interference colors. The resulting films appear naturally transparent while displaying intense colors across the visible spectrum when viewed under polarized light. We further demonstrate the universality of the iSAA strategy by fabricating films from different biopolymer fibrils (e.g., chemically modified CNFs and protein amyloid fibrils) on hydrophilic and hydrophobic substrates. Additionally, coupling the transmitted colors of these films and their intrinsic optical haze produces a novel spectrally selective polychromatic lighting effect. These results establish the iSAA strategy as a general platform for engineering nanofibril-based birefringent materials with programmable polarization optics, expanding their potential in advanced optical and energy-efficient applications.

## Introduction

Birefringent materials, which generate interference colors by inducing phase retardation of polarized light, have exhibited growing demand across diverse fields, such as information security [1–4], flexible optics [5,6], functional optical devices [7,8], and aesthetic polarization collage [9]. Interference colors, also known as birefringence colors, have been achieved beyond conventional plastics and toward the design of biopolymer materials with improved biocompatibility and biodegradability. Cellulose, with its inherent birefringence, presents a compelling choice for such optical applications, as its ordered assembly enables direct translation of molecular anisotropy into macroscopic optical effects [10,11]. Extensive efforts have been directed toward inducing long-range orientational order in cellulose nanocrystals (CNCs) to produce vivid macroscopic colors by employing alignment strategies [12]. For instance, our previous studies amplified birefringent effects by aligning CNCs within a polymer matrix through uniaxial stretching [1] and developed CNC-based heterosymmetric structures with controllable polarization optics using an evaporation-induced self-assembly (EISA) process [4]. CNC/elastomer composites exhibited stimuli-responsive colors via structural transition between chiral nematic and pseudo-nematic phases [5]. Furthermore, Ackroyd et al. [13] obtained concentric ring patterns with periodically alternating colors in Liesegang-type structures composed of CNCs and L-(+)-tartaric acid, featuring cholesteric organization and radially aligned elongated bundles, respectively.

In contrast, cellulose nanofibrils (CNFs) possess excellent tensile strength, crystal elastic modulus, and an ultralow coefficient of thermal expansion [14], and have been extensively fabricated into strong macrofibers [15,16], transparent thin films [17,18], porous membranes [19,20], and soft gels [21,22]. Specifically, fibrillated cellulose-based transparent and hazy materials have exhibited potentials for energy-efficient building applications by combining thermal insulation with uniform daylight distribution [23–25]. Notably, engineered haze films have demonstrated up to a 38% reduction in energy consumption in a model office [24]. If engineered with a macroscopic birefringent structure, CNF-derived materials could control over polarization states and phase retardation of incident light, thereby producing vibrant interference colors. Such structural engineering would couple the intrinsic properties of CNFs with advanced optical functionalities, expanding their applicability in emerging fields such as energy-efficient polychromatic illumination. However, achieving precise control over the birefringent effects remains highly challenging due to the presence of disordered amorphous domains within the nanofibril network. Unlike the vivid birefringence colors readily obtained from the alignment of CNCs, CNFs oriented by mechanical forces or external fields typically exhibit enhanced mechanical performance, anisotropic thermal conductivity, and improved biomaterial performance [26]. Additionally, the self-orienting capacity of CNFs during EISA remains insufficiently understood [27,28], and EISA-based approaches provide limited tunability over the resulting color characteristics of nanofibril patterns [29]. These limitations highlight the urgent need for new nanofibril assembly strategies capable of generating tailorable interference colors.

Striking birefringence has been found in marine zooplankton, such as ctenophores (comb jellies), medusa, pteropods, and salps [30]. For instance, the lobate ctenophore *Bolinopsis* sp. and the beroid ctenophore *Beroë ovata* display remarkable birefringence patterns originating from aligned ciliary comb rows, structured proteins, and statoliths composed of calcium carbonate [30]. Similarly, pseudothecosomatous pteropods (sea butterflies) exhibit vivid interference colors, arising from their stable, tightly packed organo–mineral shells dominated by aragonite crystals [30,31]. Inspired by these biological architectures, we hypothesize that embedding inorganic crystals within biopolymer matrices may amplify birefringence and facilitate the formation of distinct colors.

**Fig. 1.**
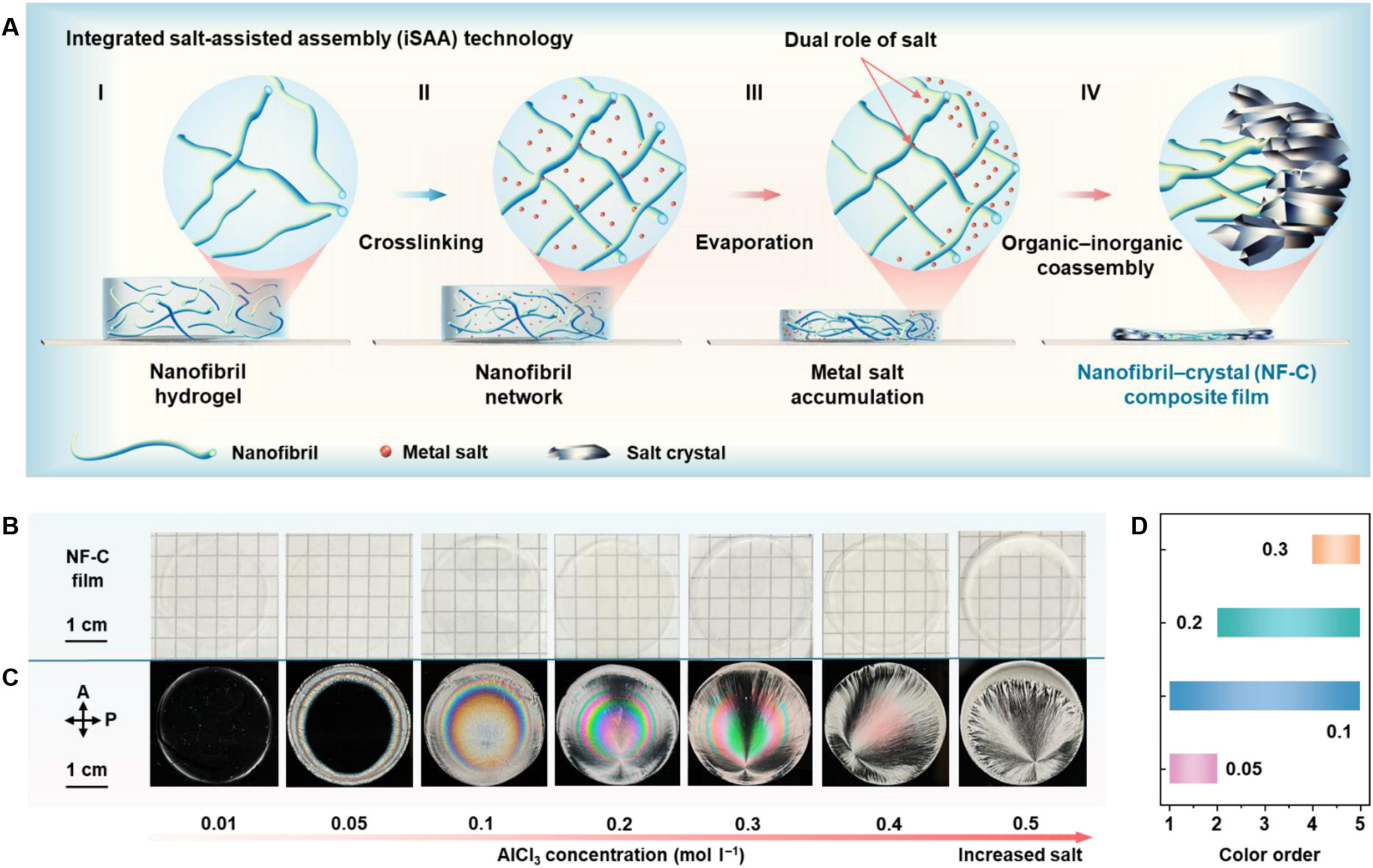
Tuning interference colors from transparent nanofibril–crystal (NF-C) composite films using an integrated salt-assisted assembly (iSAA) method. (A) Schematic illustration of the iSAA process: (I) A hydrogel composed of biopolymer nanofibrils (phosphorylated cellulose nanofibrils [PCNFs]) serves as the starting material. (II) Crosslinking with metal salt (AlCl_3_) aqueous solution. (III) Salt accumulation at the gel edge during thermal evaporation in an oven. (IV) Assembly of the NF-C composite along radial direction. (B and C) Photographs of the centimeter-scale films prepared from 0.3 wt.% PCNF aqueous suspensions crosslinked with AlCl_3_ of varying concentrations (0.01 to 0.5 mol l^–1^), as observed under natural light (B) and crossed polarizers (C). Crossed arrows indicate polarization axes of both linear polarizer (P) and analyzer (A). (D) Color order range across the Michel-Lévy color chart achieved at different AlCl_3_ concentrations (mol l^–1^).

Herein, we report an integrated salt-assisted assembly (iSAA) strategy that combines ion-crosslinking and organic–inorganic coassembly to fabricate centimeter-scale nanofibril–crystal (NF-C) composite films exhibiting vivid, tunable interference colors (Fig. 1A). The chromatic patterns are tuned by strategically selecting metal salts and optimizing process parameters including salt concentration, CNF content, and evaporation temperature. Separation of unbonded salt ions from the crosslinked gels highlights the essential effect of salts in color pattern formation. The universality of iSAA strategy is demonstrated using various biopolymers nanofibrils, such as phosphorylated and carboxylated CNFs as well as protein amyloid-like fibrils. Beyond conventional optical coding applications, we demonstrate a functionality of spectral-selective polychromatic lighting by coupling transmissive birefringence colors and optical haze of NF-C films. Overall, we establish iSAA strategy as a universal pathway for engineering nanofibril-based materials with programmable interference colors. Compared to traditional assembly processes, this approach avoids reliance on complex equipment or substrate deformability, while enabling effective color tunability and new optical and energy-efficient applications.

## Results

### Tuning of vibrant interference colors using iSAA

Phosphorylated CNFs (PCNFs) with a charge density of 2.9 ± 0.2 mmol g^−1^ is used as the starting material, and a hydrogel is obtained at a low concentration of 0.3 wt.% (P0.3), as evidenced by the higher storage modulus, *G'*, than the loss modulus, *G″*, over the frequency range (Fig. S1). Representative metal salt chlorides are employed in the iSAA approach (see Materials and Methods), with aluminum chloride (AlCl_3_) playing a key role in producing ordered, polychromatic concentric ring patterns, distinguishing it from other samples (Fig. S2). When aluminum chloride is replaced with either the sulfate or nitrate salt, the chromatic patterns still emerge in the films, although the Maltese cross is less well-constructed (Fig. S3). Additionally, increasing the concentration of PCNF aqueous suspensions enhances their elastic response and produces similar ring patterns, but leads to film cracking due to high internal stress upon drying (Figs. S1 and S4). In the following experiments, we fix the PCNF concentration at 0.3 wt.% and tailor the interference colors by varying the AlCl_3_ concentration (*c*_Al_). The resulting samples are denoted as NF-CX, where X represents the aluminum salt concentration.

As shown in Fig. 1B, all the films appear transparent without any patterns under natural light, while they exhibit isotropic, localized anisotropic, or anisotropic features between crossed polarizers, as characterized by distinct interference colors (Fig. 1C). Specifically, as the concentration increases from 0.01 to 0.05 M, a coffee ring-like pattern emerges in the outer regions, with ring colors transitioning from first-order white to second-order magenta, while the central area remains black (Fig. S5A), indicating a localized anisotropic character. At concentrations of 0.1 to 0.3 M, multiple concentric rings with vivid interference colors and regular spacing are observed. NF-C.1 displays prominent yellow, violet, and blue rings within the first 2 color orders, followed by thinner rings exhibiting colors from third-order magenta to fifth-order green (Fig. S5C). In contrast, NF-C.2 presents wider rings with colors spanning multiple orders (see details in the following section). Additionally, elevated concentration (0.3 M) results in alternating green and pink (Fig. S5E), consistent with the fourth- and fifth-order colors. The interference colors of these samples span a broad range of chromaticity coordinates within the Commission internationale de l’éclairage (CIE) 1931 color space (Fig. S5B, D, and F). As the salt concentrations increases further (*c*_Al_ ≥ 0.4 M), the formation of polychromatic ring pattern is notably suppressed, while the films still exhibit a Maltese cross.

The AlCl_3_ concentration in the range of 0.05 to 0.3 M provides a desirable window for tuning vibrant interference colors. A broad color range spanning 4 to all 5 color orders can be achieved using a moderate concentration (0.1 to 0.2 M), whereas relatively low (0.05 M) or high (0.3 M) concentrations lead to a narrower color range (Fig. 1D), highlighting the flexible color-tuning capability. Later in the text, the NF-C.2 film is selected as a representative NF-C sample for further analysis.

### Properties of NF-C composite films

**Fig. 2.**
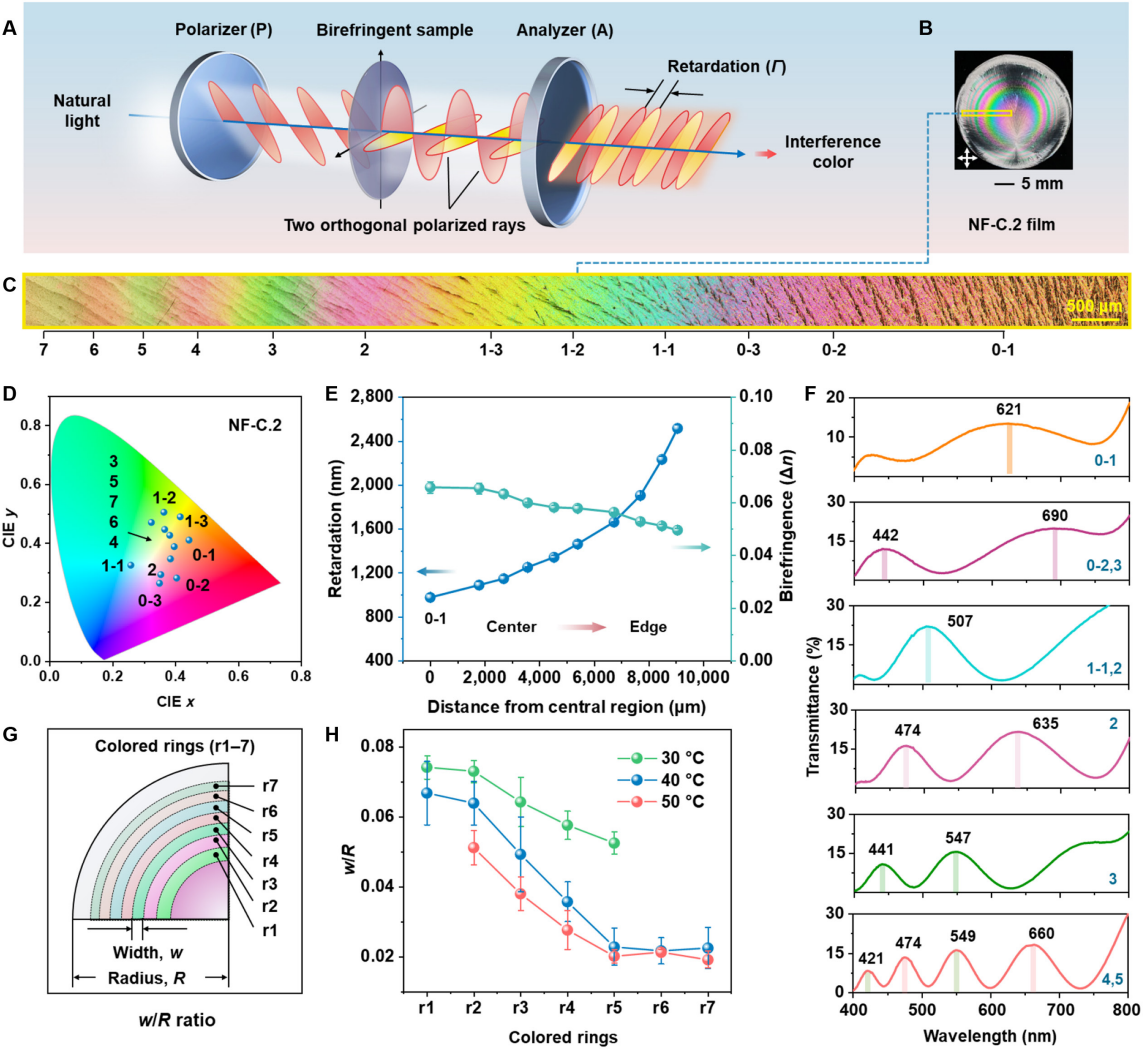
Properties of representative NF-C.2 films. (A) Schematic illustration of the mechanism underlying the formation of interference colors. When natural light passes through a birefringent material placed between crossed polarizers, phase retardation occurs between the orthogonal polarization components, resulting in constructive or destructive interference and the appearance of interference colors. (B) Photographs of a representative NF-C.2 film, as observed under crossed polarizers. The labeled area indicates the region for the polarized light microscope (POM) analysis. Crossed arrows indicate polarization axes of both linear polarizer and analyzer. (C) POM images of the labeled area in (B). The numbers below the images correspond to specific positions with distinct colors. (D) Colors of the labeled positions in (C) displaying in the Commission internationale de l’éclairage (CIE) 1931 color space. (E) Light retardation and birefringence (Δ*n*) measured from the center to the edge of the film. Error bars represent standard deviations calculated from 3 independent measurements (*n* = 3). (F) Polarized ultraviolet–visible (UV–vis) transmission spectra of the NF-C film under crossed polarizers. Black numbers indicate spectral peak position, while blue numbers correspond to the measurement positions. (G) Scheme illustrating the determination of the normalized width, represented as the width-to-radius ratio (*w*/*R*). (H) *w*/*R* values of various colored rings as a function of evaporation temperature. Note that r1 is difficult to determine from the film prepared at 50 °C. Error bars represent standard deviations calculated from 3 independent measurements (*n* = 3).

The various interference colors originate from the phase retardation, *Γ*, between 2 polarized light rays, which generate the polarization ellipse (Fig. 2A). The theoretical transmission intensity of the light (*I*) through the analyzer (i.e., intensity of transmitted light versus wavelength) is defined according to Eq. (1) [32,33]:

$$I=I_{0}\left[\cos^{2}\varphi -\sin\;2\left(\tau -\varphi \right)\;\sin2\tau \;\sin^{2}\frac{\pi \Gamma }{\lambda }\right]$$ (1)

where *I*_0_ is the intensity of the light after the polarizer, *φ* is the angle between vibration directions of the polarizer and analyzer, *τ* is the angle between the polarizer’s privileged direction and the closest privileged direction of the birefringent sample, *λ* is the wavelength of light, and *Γ* is the retardation introduced when the light passes through the sample.

When the sample is viewed 45° off extinction (*τ* = 45°) between crossed polarizers (*φ* = 90^o^), this equation reduces to Eq. (2):

$$I=I_{0}\sin^{2}\frac{\pi \Gamma }{\lambda }$$ (2)

Conversely, when the sample is viewed 45° off extinction (*τ* = 45°) between parallel polarizers (*φ* = 0°), this equation reduces to Eq. (3):

$$I=I_{0}\cos^{2}\frac{\pi \Gamma }{\lambda }$$ (3)

As shown in Fig. 2B and C, the NF-C.2 film exhibits ordered concentric rings with multiple vibrant colors, covering a large portion of the chromaticity coordinates within the CIE 1931 color space (Fig. 2D). Light retardation measurements reveal that the NF-C.2 film generates a gradient retardation ranging from 977 to 2,515 nm (Fig. 2E), corresponding to a color transition from second-order orange (position 0-1) to fifth-order green (position 5), as shown in the polarized optical microscopy (POM) images (Fig. 2C). The color distribution aligns well with the Michel-Lévy color chart [34]. Only extinction at positions 6 and 7 cannot be observed under POM when rotating the compensator due to the measurement limit of 2,800 nm. Additionally, the NF-C.1 film has relatively low retardation values ranging from 397 to 2,575 nm (Fig. S6), corresponding to broad colors spanning all orders of the chart.

To explore the origin of retardation, which is determined by birefringence Δ*n* and the optical path length (i.e., material thickness) according to Eq. (4), we further analyze the surface topography of the NF-C.2 film.

$$\Delta n=\frac{\Gamma \;}{t}$$ (4)

where *Γ* is retardation and *t* is material thickness.

As shown in Fig. S7, the film exhibits a distinct thickness variation from the center to the edge, increasing from 14.9 μm (position 0-1) to 50.7 μm (position 5). The corresponding birefringence Δ*n* values are calculated to be within the range of 0.050 to 0.066 (Fig. 2E). The variations in thickness primarily result from the nonuniform distribution of the salt crystals on the film surface, which will be discussed in the following section.

Polarized ultraviolet–visible (UV–vis) spectroscopy measurements are conducted to demonstrate the unique spectra-selective property of the NF-C.2 film. When placed between crossed polarizers, the film enables selective transmission of light, producing characteristic peaks (Fig. 2F), while the polarizers alone transmit no particular signal within the 400 to 700 nm range (Fig. S8). As the light spot moves from the center toward the edge, the peak intensity shifts accordingly. For example, at position 2, 2 peaks appear at 474 and 635 nm, which correspond to blue and red wavelengths, respectively, resulting in the observed magenta color. These experimentally measured results align well with the theoretical transmission, as calculated using Eq. (2) (Fig. S9).

In addition, thermal evaporation has a marked impact on the ring dimension and integrity of the pattern. At a low oven drying temperature, slow water evaporation allows gradual supersaturation, keeping the system close to equilibrium and promoting a slow, balanced crystallization process that yields well-ordered crystals with fewer defects. In contrast, elevated temperatures accelerate water evaporation and increase the apparent crystallization rate, but induce nonequilibrium precipitation that leads to excessive nucleation and defect-rich crystals. Specifically, as the temperature increases from 30 to 60 °C, the initially regular polychromatic rings gradually become distorted and their widths decrease (Fig. S10). Further increasing the temperature to 80 °C results in rapid evaporation and the formation of disorganized circular patterns with random colors (Fig. S10). A normalized width of each ring is defined as the ratio of the ring width to the film radius (*w/R*, Fig. 2G). In the film prepared at 40 °C (NF-C.2-40), the green ring near the center (r1) has a relatively large width with a *w/R* of 0.067, and the value then decreases steeply from 0.064 (r2) to 0.023 (r5), eventually stabilizing at ~0.02 for r5 to r7 (Fig. 2H). NF-C.2-50 exhibits slightly smaller rings but follows a similar size variation pattern relative to NF-C.2-40 (Fig. S10). In contrast, the NF-C.2-30 film shows 5 rings (Fig. S10), with moderate decreases in size from 0.074 (r1) to 0.052 (r5, Fig. 2H). We further analyze the variation in ring sizes at the same location within the films prepared at different temperatures. Taking the third green ring (r3) as an example, its *w/R* value decreases from 0.064 to 0.038 as the drying temperature increases from 30 to 50 °C (Fig. 2H). In addition, by introducing uneven heat distribution within the gel during evaporation, the resulting pattern exhibits symmetric concentric elliptical rings, with the central ellipse displaying a major-to-minor axis ratio (*a*/*b*) of 1.43 instead of a circular pattern, while the color variation trend remains consistent with that of NF-C.2 (Fig. S11).

### Salts as key determinants for pattern formation

**Fig. 3.**
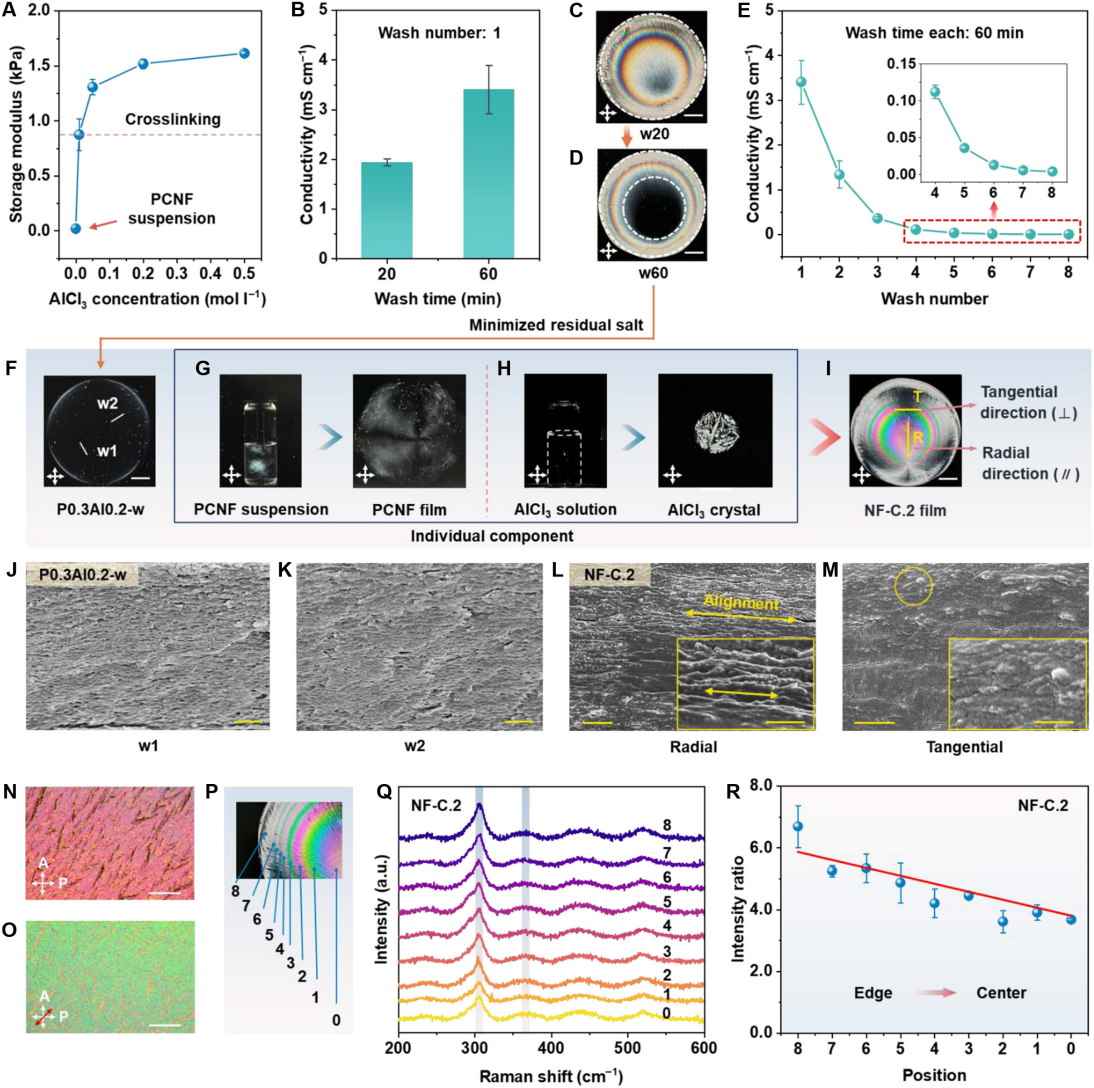
Effect of salts on pattern formation and structural features of resulting films. (A) *G'* of PCNF aqueous suspensions as a function of AlCl_3_ concentrations. (B) The conductivity of residual liquids separated from the crosslinked hydrogel after washing for 20 min (w20) and 60 min (w60). (C and D) Photographs of the w20 (C) and w60 (D) films observed under crossed polarizers. (E) Conductivity of residual liquids as a function of the wash numbers. Error bars represent standard deviations calculated from 3 independent measurements (*n* = 3). (F) The P0.3Al0.2-w film observed under cross polarized polarizers, with “w1” and “w2” indicating 2 randomly selected cross-sections. (G and H) Photographs of PCNF film prepared from a 0.3 wt.% PCNF aqueous suspension (G) and AlCl_3_ crystal formed from a 0.2 M AlCl_3_ solution (H). (I) Photograph of NF-C.2 film labeled with “R” and “T”, indicating the radial and tangential direction, respectively, for cross-sectional SEM analysis. Scale bars in (C), (D), (F), and (I) are 5 mm. (J and K) Cross-sectional SEM images of P0.3Al0.2-w film (control). “w1” in (J) and “w2” in (K) indicate 2 cross-sections of the sample. Scale bars: 1 μm. (L and M) SEM image of the cross-section along the radial (L) and tangential direction (M) of the NF-C film. The double-headed arrows in (L) indicate the orientation of the fibrils. The circle in (M) indicates the representative fibril ends. Scale bars in (L) and (M) are 1 μm and insets are 500 nm. (N and O) POM images of NF-C.2 film taken without (N) and with a λ/4 waveplate (O) inserted with the slow axis +45° relative to the polarizer. Scale bars: 250 μm. (P) Scheme showing the different positions for Raman spectra acquisition. (Q) Raman spectra collected from the edge (position 8) to the center (position 0) of the NF-C.2 film. (R) Intensity ratios of characteristic Raman peaks based on data from (Q). Error bars represent standard deviations calculated from 3 independent measurements (*n* = 3).

Upon crosslinking with AlCl_3_, *G*′ of the gel sharply increases and becomes steady at concentrations of 0.2 to 0.5 M (Fig. 3A and Fig. S12), indicating similar gel strength. Nonetheless, the films exhibit distinct polarization patterns (Fig. 1C), which suggest that unbonded salts within the cationic-bridged gels likely contribute to the observed chromatic effects. To validate this hypothesis, we design a control experiment in which the crosslinked gel is repeatedly washed to remove the remaining salts (see Materials and Methods). As shown in Fig. 3B, the residual liquid from a 60-min wash (w60) exhibits a conductivity of 3.41 mS cm^−1^, exceeding that of w20 (1.94 mS cm^−1^), which suggests increased release of free ions into the washing solution. Interestingly, the w20 film shows a chromatic pattern featuring a small isotropic region (black), which expands further in the w60 film (Fig. 3C and D). These patterns are highly similar to those fabricated from relatively low AlCl_3_ concentrations of 0.05 and 0.1 M (Fig. 1C).

After an intensified washing process, the residue conductivity drops from 3.41 to 0.0046 mS cm^−1^ (Fig. 3E), approaching that of deionized water, which indicates that free salts are nearly eliminated from the gel. Interestingly, the resulting film (P0.3Al0.2-w) appears predominantly black without vivid colors (Fig. 3F) and fails to transmit any distinct signal within the 400- to 700-nm range (Fig. S13), indicating an isotropic feature that contrasts with NF-C.2 (Fig. 2B and C). Raman spectra reveal that only cellulose bands are detected in P0.3Al0.2-w (Fig. S14 and Note S1), confirming the removal of residual salts, which aligns well with the conductivity analysis. Although the removal of unbonded salt suppresses color formation, the films prepared from the gels after washing exhibits high tensile strengths (Fig. S15). In contrast, the deposition of more salt crystals reduces flexibility of the composite films.

Additionally, neither the neat PCNF films nor the salt crystals themselves exhibit vivid interference colors. The PCNF films display only weak birefringence (Fig. 3G), while the AlCl_3_ crystals feature a disordered, multibranching dendritic structure (Fig. 3H). This finding highlights the critical role of additional unbonded salts in generating organized chromatic pattern absent in the individual components (Fig. 3I), where the NF-C composite synergistically assembles along the radial direction, as discussed later. We further estimate the residual salt content within the gel by inferring the salt concentration of each collected residual liquid from its conductivity using the conductivity–concentration calibration curve (Fig. S16). As a result, the predicted weight ratio of aluminum salt relative to PCNFs in NF-C.2 films is approximately 5.7, corresponding to 1.7 wt.% of salt relative to the total hydrogel weight.

### Morphological and structural features of the NF-C.2 film

Cross-sectional scanning electron microscope (SEM) analysis of P0.3Al0.2-w is performed at 2 randomly selected positions (Fig. 3F), while NF-C.2 is observed at specific radial and tangential directions (Fig. 3I). As a result, P0.3Al0.2-w exhibits similar layered structures in 2 cross-sections (Fig. 3J and K). In contrast, the fracture along the radial direction of NF-C.2 exhibits tightly packed fibril bundles (Fig. 3L). Additionally, in the cross-sectional images from tangential direction (i.e., perpendicular to the radial direction), individual fibrils appear as short, pulled-out structures resembling multiple white dots, which are widely dispersed at a scale of approximately 50 nm (Fig. 3M). Representative individual fibril entities are highlighted with a yellow circle. Similar structural features have also been reported in aligned cellulose fibers fabricated via a flow-focusing spinning method [16]. Surface SEM images of control samples further reveal a dense surface with random morphologies and no observable salt crystals (Fig. S17A). The surface is covered with aluminum chloride hexahydrate crystals (Fig. S17B), as confirmed by the x-ray diffraction (XRD) pattern (Fig. S18). Fracture and surface morphologies verify the anisotropic property and radial orientation of the NF-C assemblies. Furthermore, when inserting a quarter-wave plate, the observed color shifts from second-order magenta to third-order green (Fig. 3N and O), indicating an increase in the light retardation. This phenomenon confirms the radial orientation of the composite, aligning with the SEM analysis.

To analyze the distribution of the salt crystal, we further perform Raman measurements at positions 0 to 8, covering distinct rings as well as the center and edge of the NF-C.2 film (Fig. 3P). As shown in Fig. 3Q, Raman spectra acquired from various regions exhibit characteristic bands of AlCl_3_·6H_2_O crystals, with a prominent peak emerging at 306 cm^−1^. The peak intensity ratio of salt to cellulose (*I*_306_/*I*_366_) gradually decreases from 6.7 to 3.7 (Fig. 3R), suggesting reduced salts from the edge to the central region. Such a nonuniform distribution of the salt crystals contributes to thickness variations in the films (Fig. S7), as described above. In contrast, the P0.3Al0.2-w film, which lacks crystal deposition, exhibits a uniform thickness of approximately 7 μm.

### Proposed iSAA mechanism for the pattern formation

In conventional salt-assisted assembly (SAA), salt ions primarily serve as passive charge screeners, reducing electrostatic repulsion between charged building blocks and thereby enabling electrostatically driven assembly. By contrast, our iSAA strategy utilizes salts as active drivers of crosslinking and organic–inorganic coassembly, producing birefringent architectures with tunable interference colors.

**Fig. 4.**
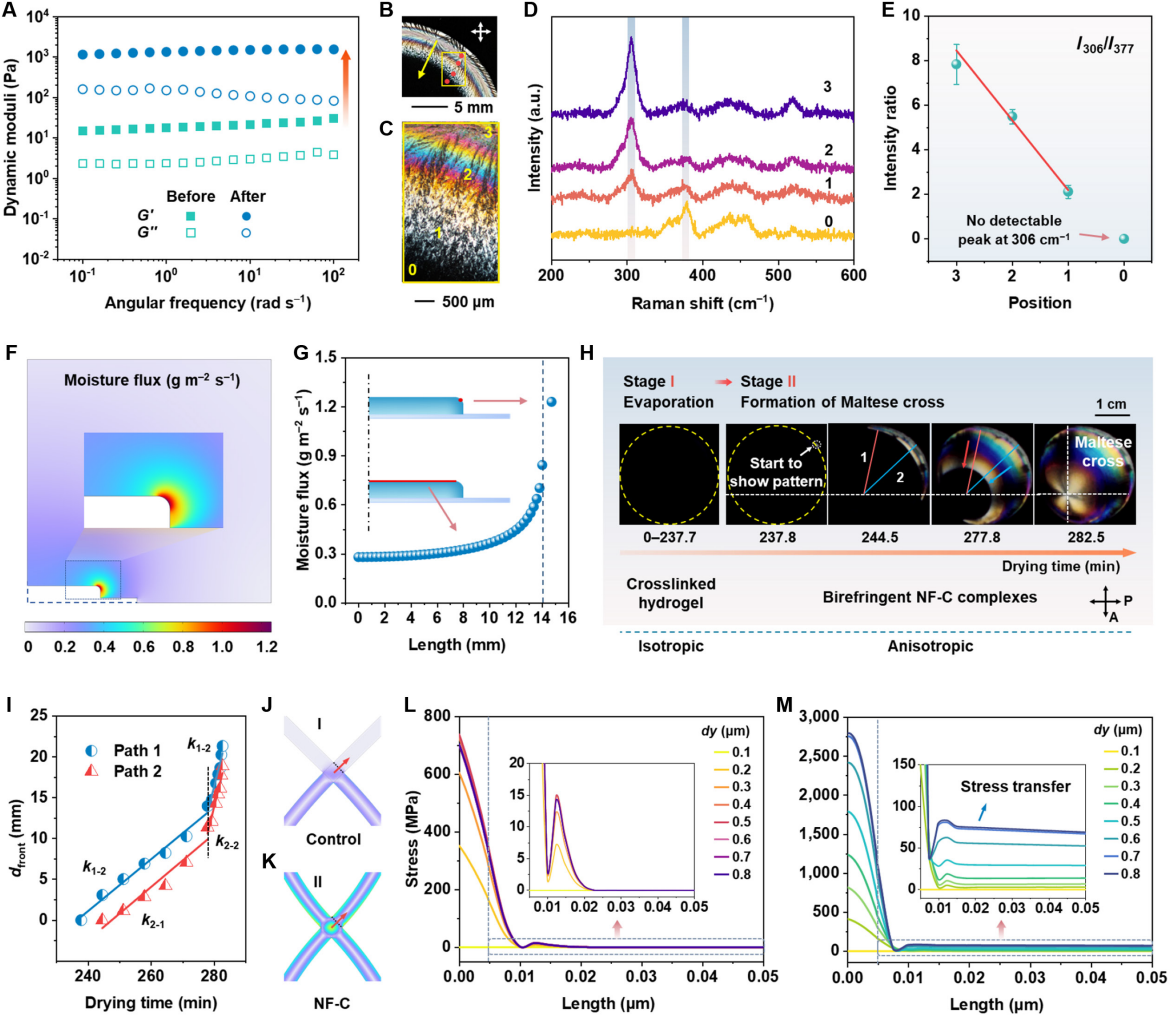
Mechanism of the formation of chromatic pattern from the iSAA process. (A) Dynamic moduli (*G′* and *G″*) of 0.3 wt.% PCNF aqueous suspensions before and after crosslinking with AlCl_3_. (B) Photograph of the NF-C film prepared with 0.05 mol l^–1^ AlCl_3_ solution (NF-C.05), observed between crossed polarizers. (C) POM image of the labeled area in (B). The numbers indicate specific positions selected for Raman analysis. (D) Raman spectra acquired from positions 0 to 3 of the NF-C.05 film. (E) Peak intensity ratio of AlCl_3_·6H_2_O (306 cm^−1^) to cellulose (377 cm^−1^). Error bars represent standard deviations calculated from 3 independent measurements (*n* = 3). (F) Numerical simulations of 2D moisture flux distribution over the gel surface after evaporation at 40 °C for 60 s. (G) Numerical calculation of moisture flux. The red line and point in the insets indicate the position where data are acquired. (H) Snapshots of the sample taken at different drying times reveal a disorder-to-order transition. This evolution proceeds from thermal evaporation of the crosslinked hydrogel in an isotropic state (Stage I) to the formation of a characteristic Maltese cross (Stage II), as drying progresses. (I) Displacement of the drying front (*d*_front_) of the NF-C composite as a function of drying time during the formation of characteristic Maltese cross (stage II). The blue and red arrows direct to the drying front along the path lines 1 and 2, respectively. (J and K) Magnified images highlighting stress distribution in the central areas of the nanofibril network control (J) and NF-C (K). (L and M) Numerical calculation of stress distribution along the center-to-top-right direction of the nanofibril network blank control (L) and NF-C (M) as a function of transfer length, with crystal displacement from 0.1 to 0.8 μm in the *y* direction. The dashed line marks the fibril intersection.

The 0.3 wt.% PCNF aqueous suspensions exhibit gel-like behavior before salt addition (Fig. 4A) and exhibit birefringence under crossed polarizers (Fig. 3G) because the individually dispersed nanofibrils spontaneously align into a locally nematic-like order [35]. Surface charges (e.g., phosphate groups) result in electrostatic interactions, and the system achieves a balance between electrostatic repulsion and attractive forces, contributing to the stable dispersion of oriented nanofibrils [35,36]. The birefringence is retained in the resulting PCNF thin film (Fig. 3G). In contrast, crosslinking with aluminum salts yields a gel that appears dark under crossed polarizers, indicating an isotropic nanofibril network. After removing unbonded salts, the resulting P0.3Al0.2-w films retain this isotropic optical response (Fig. 3F). Note that this behavior suggests that ion-bridged nanofibril networks resist capillary-force-driven spontaneous orientation during solvent evaporation, in contrast to the alignment typically induced by EISA [12].

The transition from local anisotropy to isotropy in CNF aqueous suspensions differs from the salt-induced nematic liquid crystalline behavior observed in CNC systems [37–39]. Typically, the addition of salts (e.g., NaCl and CaCl_2_) can trigger early CNC aggregation by enhancing intermolecular attraction due to charge screening, and it facilitates a nematic phase transition prior to complete aggregation because lateral orientation represents the most energetically favorable state. The transition regime is governed by an equilibrium of repulsive and attractive forces within the suspension [39]. In contrast, our analysis suggests that in CNF aqueous suspensions, salt addition primarily leads to the formation of a confined nanofibril network through cationic bridging, and unbonded free salts play a dominant role in guiding the assembly process, as demonstrated above. Specifically, we propose that the development of the chromatic polarization pattern occurs through 3 key steps: (a) ion crosslinking of CNF hydrogels, (b) accumulation of metal salt at the gel edge during evaporation, and (c) crystal growth-induced stress transfer for NF-C coassembly along the radial direction.

In the first step, the *G*′ values of the crosslinked gels upon introduction of Al^3+^ are 2 orders of magnitude higher than those of the PCNF aqueous suspensions (Fig. 4A), indicating the formation of a stronger interconnected network due to enhanced interfibrillar cohesive interactions [22]. These strong interactions primarily arise from the phosphate–aluminum complexation, as confirmed by Fourier transform infrared (FTIR) and x-ray photoemission spectroscopy (XPS) analyses (Fig. S19). In the FTIR spectra of the crosslinked samples, the band at 1,232 and 920 cm^−1^, corresponding to the P=O and P–OH stretching mode [40], exhibits reduced intensities, while the P–O–C band shifts from 824 to 835 cm^−1^ (Fig. S19A). Furthermore, as shown in the XPS spectra, PCNFs exhibit peaks at around 133 eV (Fig. S19B), which is assigned to P 2p signals [41]. High-resolution scans indicate that the P 2p peak shifts from 133.1 eV in pristine PCNFs to 133.5 eV after crosslinking (Fig. S19C). Metal–ligand coordination bonds arise from the donation of lone-pair electrons to the vacant orbitals of metal ions, leading to reduced local electron density at the ligand sites and an increase in the corresponding core-level binding energy [42,43]. In this work, the phosphate oxygen atoms donate lone-pair electrons to the electron-deficient Al^3+^ centers, forming phosphate−aluminum coordination bonds. This electron donation reduces the electron density of the P–O bonds and consequently lowers the local electron density around phosphorus, resulting in an increased P 2p binding energy. Previous studies have also reported an increase in the N 1s binding energy associated with reduced local electron density at nitrogen atoms due to amino–metal complexation [42,43]. The effective cationic bridging of nanofibrils establishes a well-preserved, strong gel network for the subsequent assembly of the composite.

To investigate the nonuniform distribution of metal salts within the gel network during evaporation, we analyze the surface pattern of the NF-C.05 film that shows localized anisotropy (Fig. 4B and C). Raman spectra reveal a substantial decrease in the AlCl_3_·6H_2_O peak intensity from the film edge toward the center (Fig. 4D), quantified by a drop in the intensity ratio (*I*_306_/*I*_377_) from 7.83 at position 3 to 2.21 at position 1 (Fig. 4E). In the internal region, the ratio is considered zero due to the absence of a detectable peak at 306 cm^−1^ (Fig. 4E). These results show that salt crystals preferentially accumulate at the edge rather than in the central region.

The uneven salt distribution can be attributed to the outward flow of ions driven by capillary forces during evaporation [44,45], which arises from gradient in moisture flux along the gel surface (Fig. S20 and Fig. 4F). Specifically, the flux gradually increases from 0.28 to 0.45 g m^−2^ s^−1^ near the edge (*L* = 12 mm), then sharply rises to 1.23 g m^−2^ s^−1^ at the position marked by a red dot (Fig. 4G). As evaporation proceeds, the gel will present a morphology resembling a sessile droplet, with a contact angle of less than 90°. To minimize interfacial area, surface tension generates a capillary flow that drags suspended salt ions toward the edge, leading to their accumulation and the formation of a concentration gradient. The evaporation-driven internal flow is associated with the well-known coffee-ring effect, where solid particles in a droplet suspension preferentially deposit near the pinned contact line during evaporation [44,45]. Similar effects have been observed in crystallizing solutes, including simple salts, proteins, polyelectrolytes, and their mixtures [45]. In our system, the salt accumulation and elevated moisture flux near the gel edge both accelerate supersaturation, promoting inward crystal growth along the radial direction of the circular sample and the corresponding ordered assembly.

By in situ monitoring the hydrogel evaporation (Fig. S21), we suggest that the entire process includes transition from a black appearance due to a randomized fibrous network to a color pattern resulting from a well-organized structure (Fig. S22 and Movie S1). The evolution of chromatic polarization pattern can be divided into 4 stages: water evaporation of the crosslinked hydrogel (stage I), formation of characteristic Maltese cross (stage II), emergence of homogeneous colors (stage III), and development of colored rings (stage IV). In particular, we focus on stage II, where the order-to-disorder transition starts, and track the growth dynamics of the drying front. As evaporation progresses, a brilliant pattern emerges from the periphery, and the drying front showing vivid interference colors moves radially toward the central area, eventually forming a characteristic Maltese cross (Fig. 4H). The drying front experiences a slow process (~40 min) with a slope of 0.32 mm min^−1^ (*k*_1-1_), followed by a fast process (~4.7 min) with a steeper slope of 1.53 mm min^−1^ (*k*_1-2_) before forming a Maltese cross (Fig. 4I). A similar trend is observed along path 2 (*k*_2-1_ = 0.33 mm min^−1^, *k*_2-2_ = 1.57 mm min^−1^).

The components of the films along the radial direction are CNF/crystal composite, as confirmed by Raman spectra (Figs. 3Q and 4D). POM and SEM analyses (Fig. 3L to O) further suggest that the colored drying front is composed of radially oriented CNF/crystal structures, and its movement reflects an ongoing assembly process. The assembly mechanism is similar to the ice-templating effect, where ice crystals grow along a temperature gradient, anchoring one end of the CNFs, expelling them, and exerting shear forces between the fibrils and adjacent crystals, thereby promoting fibril orientation [26,46]. Similarly, in our system, early crystallization at the edge stabilizes PCNFs at one end. As salt crystals grow inward along a concentration gradient arising from salt accumulation and moisture flux difference, they expel and shear the nanofibrils, ultimately forming a birefringent CNF–crystal composite responsible for the vivid interference colors. Additionally, ion bridging facilitates the formation of a well-preserved, crosslinked fibril network, serving as an essential prerequisite for the organized assembly.

To evaluate the behaviors of the nanofibrils upon contacting with the crystals, we perform a contact mechanics finite element analysis (FEA) based on a 2-dimensional (2D) model (see Materials and Methods). The simulation results indicate the effective orientation of the fibril components in the NF-C network along the *y* direction, as the crystals advance by 0.8 μm. The control group remains unchanged at 45° (Fig. S23A), while the internal fibrils of NF-C network are oriented at 75°, approaching the principal direction (90°) (Fig. S23B). This difference arises from variations in stress transfer within these 2 networks, where the control shows uneven internal stress distribution with nearly zero upward transfer (Fig. 4J), while the NF-C network exhibits a more symmetric stress profile (Fig. 4K). Furthermore, we quantify the stress transferred from the center to the top right of the network along a 0.05-μm path (Fig. 4L and M). As the crystals move inward (*dy* = 0.8 μm), the stress in the control group reduces to zero along the path after a slight increase to 14.28 MPa at 0.012 μm. For the NF-C network, the stress initially drops to a minimum of 41.01 MPa after crossing the fibril intersection and then gradually increases, stabilizing within the range of 68 to 75 MPa. Similar stress transfer trends within the networks are observed throughout the entire process.

Based on these analyses, we suggest that during the iSAA process, ion crosslinking of CNF hydrogels via phosphate–aluminum coordination bonds establishes nanofibril networks. During subsequent evaporation, a gradient in moisture flux along the gel surface facilitates capillary force-driven outward ion transport, leading to salt accumulation at the gel edge. This accumulation initiates inward, directional salt crystallization from the periphery toward the center. As the salt crystals grow, they expel and exert shear forces on the nanofibrils, enabling efficient stress transfer within the crosslinked network and triggering synergistic coassembly of the NF-C composite. The resulting radially organized composite structure amplifies macroscopic birefringence and introduces thickness variations, thereby leading to controlled light retardation and vivid interference colors.

### Generality of iSAA approach

A universal fabrication method can be applied to a wide range of materials to enable diverse application potentials. To demonstrate the generality of our iSAA strategy, we apply it to 3 representative biopolymer nanofibrils: PCNFs and 2,2,6,6-tetramethylpiperidine-1-oxyl (TEMPO)-oxidized CNFs (TOCNFs), as well as bovine serum albumin (BSA) amyloid fibrils.

**Fig. 5.**
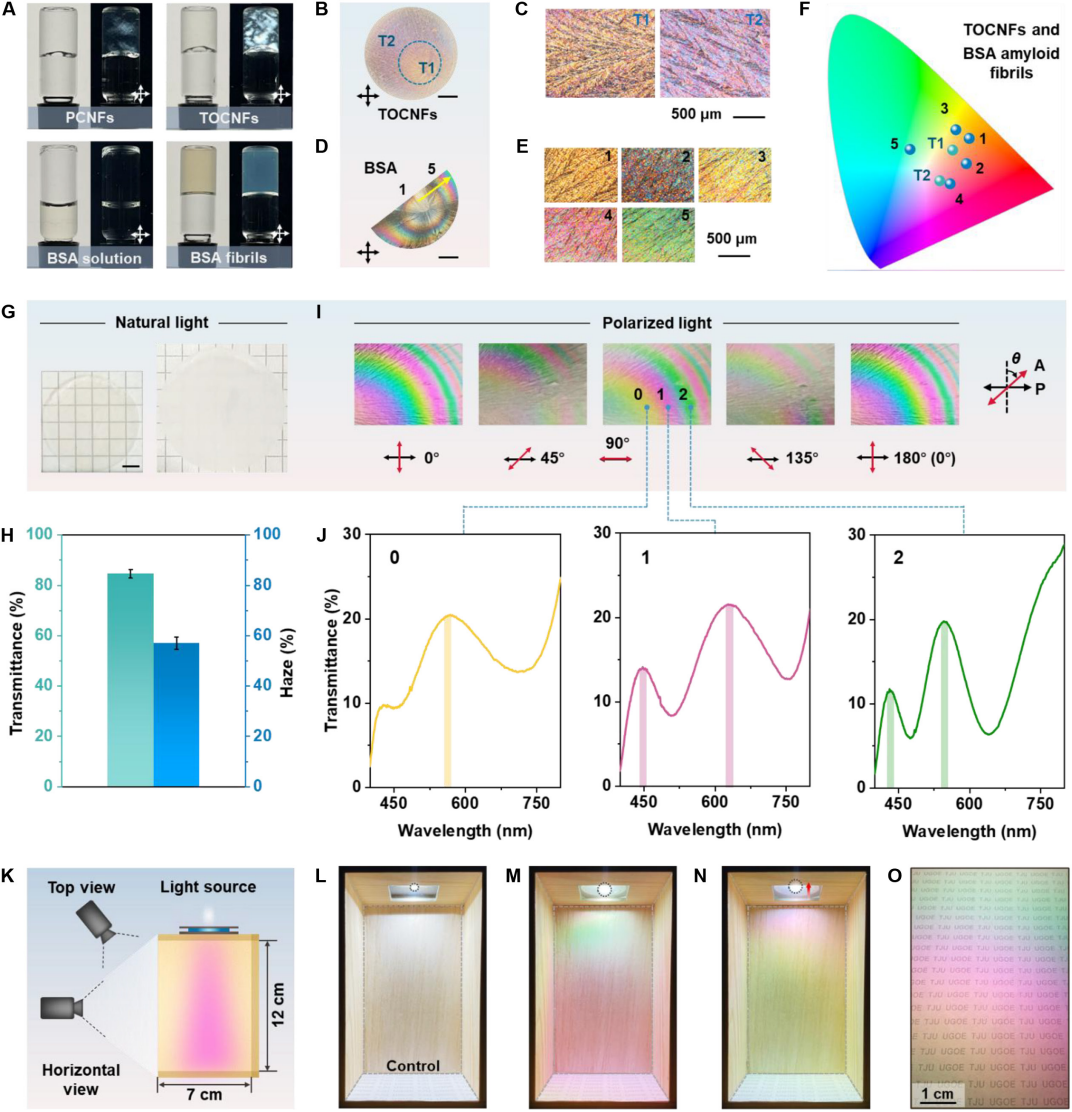
Demonstration of generality of iSAA and application potential for polychromatic lighting. (A) Photographs of PCNFs (0.3 wt.%), TEMPO-oxidized CNFs (TOCNFs) (1.0 wt.%), bovine serum albumin (BSA) aqueous solution (4 wt.%), and BSA amyloid fibrils. (B and D) Photograph of composite films with inference colors from TOCNFs (B) and BSA amyloid fibrils (D) viewed between crossed polarizers. Scale bars: 5 mm. (C and E) POM images of the labeled positions of TOCNF-based (C) and BSA-based (E) composite film. (F) Colors of the labeled positions in the films displaying in the CIE 1931 color space. (G) Photographs of the NF-C film placed directly on the substrate (left) and 3.5 cm above the substrate (right). Scale bar: 5 mm. (H) Light transmittance and haze of the NF-C film. Error bars represent standard deviations calculated from 3 independent measurements (*n* = 3). (I) Changes in the chromatic pattern of NF-C films when the analyzer is rotated 180° in steps of 45°. *θ* indicates the rotation angle of analyzer (A). (J) UV–vis transmission spectra of the different positions of the NF-C film under parallel polarizers. (K) Experimental setup for polychromatic lighting measurement under horizontal and top views. (L to N) Photographs of model rooms installed with (M and N) the NF-C film when moving the white light spot inward compared to (L) the control. (O) A top-view photograph showing the colored substrate placed at the bottom of the model room.

The 0.3 wt.% TOCNF aqueous suspension (T0.3) appears liquid-like (Fig. S24, inset) and shows much lower viscosity over the shear rate range compared to the P0.3 sample (Fig. S24). Upon crosslinking with 0.2 M of AlCl_3_, it forms a wrinkled gel with small overlapping bulk regions (Fig. S25A). The resulting films exhibit dendritic structures and irregular local coloration, rather than ordered ring patterns (Fig. S25B). Additionally, increasing the TOCNF concentration to 1 wt.% leads to a gel-like sample with intrinsic birefringence (Fig. 5A) and shear-thinning behavior comparable to those of P0.3 (Fig. S24). The corresponding TOCNF-based composite film exhibits a central orange circle (T1) and a wide ring (T2) featuring a purple interference color (Fig. 5B and C). Furthermore, protein nanofibrils also serve as effective starting materials for the color formation. As shown in Fig. 5A, BSA aqueous solution appears mostly black under crossed polarizers. After a straightforward disulfide reduction step induced by tris(2-carboxyethyl) phosphine hydrochloride (TCEP), amyloid fibril-based hydrogels form and show birefringence under polarized light (Fig. 5A). The resulting film exhibits concentric rings with interference colors ranging from first-order yellow to third-order green (Fig. 5D and E). Due to the brittle nature of the protein nanofibril-based films, cracking occurs during drying, leaving only approximately half of the films intact. While improving the film flexibility is beyond the scope of this work, previous studies have demonstrated how this could be achieved with plasticizing additives (e.g., glucose [47]). These interference colors from TOCNF- and BSA amyloid fibril-based films are mapped in the CIE 1931 color space, revealing a broad color range (Fig. 5F). The successful fabrication of these centimeter-scale samples with ordered chromatic pattern highlights the generality of the iSAA approach. Interestingly, the film prepared using positively charged chitin nanofibrils as the starting material exhibits an irregular chromatic pattern (Fig. S26), indirectly highlighting that a well-preserved, crosslinked nanofibril gel network is a crucial prerequisite for the formation of an organized chromatic pattern. Furthermore, the sample based on molecular-scale cellulose phosphate shows rigid block-like appearances, lacking ordered polychromatic patterns (Fig. S27).

In addition to varying starting materials, we perform our iSAA method using distinct substrates. As shown in Fig. S28, a range of materials spanning hydrophilic to hydrophobic, such as glass, polyethylene terephthalate (PET), and polytetrafluoroethylene, serve as excellent substrates for producing the polychromatic surface patterns, revealing a broad applicability. Furthermore, polychromatic NF-C films exhibit areas of approximately 680 mm^2^, which is 1 to 2 orders of magnitude larger than those of birefringent films reported in previous studies (Table S1), highlighting the potential of the iSAA technique for large-area processing. Moreover, iSAA exhibits favorable reproducibility, as confirmed through the fabrication of 3 representative centimeter-scale NF-C films (Fig. S29).

### Application potential in spectra-selective polychromatic lighting

Cellulose-based optical materials have attracted increasing attention for versatile applications. For instance, CNC-immobilized cellulose derivatives with aromatic substituents (CX@CNC) exhibit ultralong room-temperature phosphorescence and have been used as eco-friendly security inks for anticounterfeiting and information storage [48]. A lignin capturing–fusing strategy has also been developed to fabricate optical biofilters with near-infrared transparency for fruit detection and information protection [49]. Hydroxypropyl cellulose (HPC)-based cholesteric liquid crystal fibers fabricated by direct drawing show tunable structural colors and multifunctional properties, including self-healing, electrical conductivity, and thermal sensing [50]. Moreover, birefringent nanocellulosic materials have primarily been explored for optical coding and information encryption potentials (Table S1). In contrast, this work integrates transmissive birefringence colors with optical haze in NF-C films to demonstrate a new functionality of spectrally selective polychromatic lighting. Specifically, black lines are visible when the film is on the substrate but disappear when raised by 3.5 cm (Fig. 5G), indicating high transparency and strong forward scattering, as further confirmed by the transmittance of 84.6% and haze of 57.0% (Fig. 5H). We further investigate the spectra-selective polychromatic lighting performance of the NF-C.2 film. As shown in Fig. 5I, the surface pattern of NF-C.2 undergoes marked changes when the analyzer is rotated. Notably, when the privileged directions of the polarizer and analyzer are parallel to each other, the transmitted spectra are inverted compared to those observed under crossed polarizers, resulting in the appearance of complementary colors (Fig. 5I). For example, the magenta ring (r1) area exhibits a spectrally selective transmittance spectrum with 2 peaks at 448 and 628 nm (Fig. 5J), while the same position of the film under crossed polarizers shows a green color (Fig. 5I). The characteristic peak positions of the curve align well with the theoretical values, as determined using Eq. (3) (Fig. S30). In comparison, the parallel polarizers transmit the light without particular peaks in the range 400 to 700 nm (Fig. S8).

As a proof-of-concept demonstration of polychromatic lighting, the NF-C.2 film is sandwiched between 2 parallel polarizers and the model room is observed from both horizontal and top views as the light source moves inward (Fig. 5K). As a result, the room appears brilliant white when illuminated with white light through glass (control group, Fig. 5L). In contrast, when passing through the NF-C.2, the white-light illumination becomes polychromatic with green, magenta, and yellow being distinctly observed (Fig. 5M and N and Movie S2). Additionally, the substrate featuring words “UGOE” and “TJU” appears iridescent from a top view (Fig. 5O and Movie S3). These colors correspond to the selective transmittance spectra (Fig. 5J).

The spectrally selective polychromatic lighting enabled by the NF-C.2 films offers promising application potential in various fields, such as horticulture, agriculture, and interior design. For example, red (600 to 700 nm) and blue (400 to 500 nm) light, which correspond to the absorption peaks of chlorophyll, are the most crucial wavebands for driving photosynthesis [51]. Increased lettuce yield over 20% has been achieved by a unidirectional light-extracting fluorescent film that can enhance the proportion of red light [51]. Additionally, haze materials are expected to reduce indoor energy consumption in model offices by up to 38%, supporting the development of green buildings [24]. Consequently, by harnessing daylight, the NF-C.2 films offer a potential energy-efficient solution for colorful lighting management of crops, with the promise of reducing reliance on artificial light-emitting diode (LED) lighting. Regarding practical applicability, the birefringent NF-C samples are primarily fabricated as thin coatings on solid substrates (e.g., glass and PET sheet) rather than as free-standing materials. These film coatings exhibit strong adhesion to the substrates and form stable layered structures, thereby mitigating concerns related to their mechanical strength during use. Additionally, to display interference colors, the films are typically sandwiched between a polarizer and an analyzer, which also function as protective barrier layers that reduce direct moisture exposure and help maintain optical performance. Moreover, the iSAA-based fabrication of NF-C films provides sustainability advantages, including biodegradable starting materials, low material costs (~0.12 $ m^–2^, Tables S2 and S3), and a straightforward processing route, making this system highly attractive for practical applications.

## Discussion

Inspired by natural microstructures, such as marine zooplankton, we embed salt crystals within bio-based nanofibril matrices to enhance birefringence. The resulting self-organized NF-C composites manipulate light retardation to produce vibrant interference colors that span multiple orders of the Michel-Lévy chart, demonstrating the broad color-tuning capability of the iSAA strategy. Nanocellulose-based interference color design relies on common assembly strategies, such as external electric or magnetic fields, mechanical stretching, and EISA. However, these methods face inherent limitations including the need for complex equipment, restrictions on substrate deformability, or limited color tunability [52]. In contrast, the iSAA approach establishes organic–inorganic assembly as an alternative pathway for controllable polarization optics, expanding the accessible building blocks from nanorods to high-aspect-ratio nanofibrils while overcoming the limitations of traditional processes. Moreover, this method redefines the role of salt from a passive trigger for electrostatic screening or templating in typical SAA systems into an integrated driver for crosslinking and assembly, enhancing structural order and optical anisotropy.

From an application perspective, the combination of transmissive birefringence colors and optical haze in NF-C films enables spectral-selective polychromatic lighting, extending beyond typical information encryption applications. By harnessing natural light, these films have the potential to reduce energy demand for artificial electric lighting (e.g., LEDs), offering a sustainable colorful lighting strategy that may further reduce greenhouse gas emissions and decrease reliance on scarce or toxic materials. Potential applications include agriculture, where optimized spectral transmission could improve crop yields, and interior design, such as retail and art exhibitions, where tailored lightning can influence human emotions and cognition. In future studies, the fabrication of free-standing NF-C films with a controlled balance among mechanical strength, environmental tolerance (e.g., moisture stability), and optical performance represents an attractive direction. Composite design strategies, such as encapsulation of NF-C films within elastomeric matrices, provide a promising route toward this objective. Further manufacturing up-scaling could be achieved using bar-coating or industrially viable roll-to-roll processes. Defect-free film formation requires precise control of feed-suspension rheology for uniform wetting and spreading, as well as optimized mass and heat transfer during crosslinking and drying. Overall, this work establishes iSAA as a universal strategy for engineering nanofibril-based birefringent materials with programmable polarization optics, thereby enabling advanced optical and energy-efficient applications.

## Materials and Methods

### Materials

BSA (Fraction V) with a molar mass of ~66,000 g mol^−1^ and aluminum sulfate octadecahydrate (≥98%) were purchased from Carl Roth (Germany). Sodium chloride (≥99%), magnesium chloride (anhydrous, ≥98%), iron(III) chloride hexahydrate (≥98%), aluminum chloride hexahydrate (99%), aluminum nitrate nonahydrate (≥98%), and TCEP (powder) were supplied by Sigma-Aldrich. Calcium chloride (dried powder, 97%) was provided by Thermo Fisher Scientific. All chemicals were used without purification. Deionized water was used in all experiments.

### Preparation of bio-nanofibrils

PCNFs were prepared according to our previous studies [53]. In brief, the raw cellulose pulp was soaked in NH_4_H_2_PO_4_/urea mixture (anhydroglucose units/NH_4_H_2_PO_4_/urea = 1:0.6:3.2) at 80 °C for 25 min, followed by oven drying at 105 °C until a constant weight was reached, and then cured at 150 °C for 20 min. The cellulose carboxylation was achieved using the TEMPO/NaBr/NaClO reaction system [54]. The pH of the mixture was maintained at 10 during reaction until no NaOH consumption was observed. The obtained phosphorylated and TEMPO-oxidized fibers were thoroughly washed and subsequently subjected to mechanical disintegration to produce nanoscale fibrils. Additionally, BSA amyloid fibrils were prepared based on the previous work [55]. Briefly, BSA was dissolved in deionized water to form a solution with a concentration of 40 mg ml^–1^, followed by mixing with TCEP solution (40 mg ml^–1^). The volume ratio of TCEP/BSA was 0.06. The mixture was allowed to stand still at room temperature to form hydrogel.

### Fabrication of NF-C composite films

PCNF hydrogel was soaked in metal salt solutions at a ratio of 1:2 (w/v), followed by continuous shaking at 100 rpm for 24 h using an RS-OS 6 M shaker (Phoenix Instrument GmbH, Germany). Then, the crosslinked gel was transferred onto a glass substrate (root mean square roughness < 1 nm) and dried in an oven by natural convection without activating the fan (Memmert GmbH, Germany). Representative alkali metal (Na), alkaline earth metal (Mg and Ca), transition metal (Fe), and posttransition metal (Al) chlorides were employed, with processing parameters, including CNF content (0.2 to 0.5 wt.%), salt concentration (0.01 to 0.5 mol l^−1^), and drying temperature (30 to 80 °C), designed to tuning the interference colors.

### Removal of unbonded salts for a control sample

The control sample was prepared by separating excess unbonded salts from the crosslinked hydrogel (P0.3Al0.2) via repeated washing, followed by oven-drying at 40 °C. The conductivity of the residual liquids separated from the gel was measured by an 856 Conductivity Module (Metrohm, Germany) to monitor the washing process and estimate the remaining salt contents within the gel. The initial salt agents (~5 ml) were removed before washing, and the crosslinked gel was promptly rinsed 3 times with fresh deionized water (5 ml) to remove the residues from the gel surface. The washing process involved immersing the gel in 5 ml of deionized water under shaking at 100 rpm and was divided into 2 groups: (a) Fixed wash number: the gel was washed once for 20 min (w20) or for 60 min (w60); (b) Fixed wash time interval: the gel was washed 8 times, with each wash lasting 60 min. After each wash, the same cleaning procedure described above was followed, and the residual liquid (20 ml) was collected into a glass vial for conductivity measurements. Furthermore, to estimate the salt content in residual liquids, a known amount of AlCl_3_·6H_2_O was dissolved in deionized water and the solution’s conductivity was measured at 25 °C to establish a concentration–conductivity calibration curve. The AlCl_3_ concentrations ranged from 0.0001 to 0.02 mol l^–1^.

### FEA of hydrogel evaporation

Moisture flux above a hydrogel at an initial temperature 40 °C and a relative humidity of 30% was simulated using COMSOL Multiphysics. The geometrical model was constructed according to the experimental parameters (Fig. S31). The laminar flow and moisture transport in air interfaces were utilized, and the wet surface feature was assigned to the gel surface to implement the source term for the water vapor. Details are presented in Note S2.

### FEA of crystal growth-induced stress transfer within nanofibril network

Nucleation and crystal growth are 2 essential processes in the formation of salt crystals. During the process, salt ions start to aggregate into nuclei in a supersaturated solution, and additional ions attach to these nuclei, resulting in the development of larger crystals. To simplify the simulation, the displacement of the geometrical models was used to simulate the crystal growth in this system. The structural contact in the Solid Mechanics interface was employed to analyze the stress distribution and deformation of the nanofibril network upon contact with the crystals.

Model geometries including CNFs (length: 1 μm, width: 10 nm) and aluminum salt crystals were built up (Fig. S31). Note that only part of the crystal was modeled for the contact. All fibrils were oriented at ± 45°, forming a network with a 2D orientational order parameter (S_2D_) value of zero, thereby enabling a clear observation of deformation upon interaction with the crystals. Specifically, the fibril network with internal fibrils disconnected from the upper adjacent ones was designed to simulate locally uncrosslinked fibril network and served as a control group (Fig. S32A) for comparison with the intact network (Fig. S32B). The bottom components were set as fixed constraint to simulate the pinned fibrils during film formation. For material assignment, CNFs are defined as linear elastic materials with a density of 1.6 g cm^−3^, an elastic modulus of 80 GPa, and a Poisson’s ratio of 0.38, while AlCl_3_·6H_2_O crystals are modeled as rigid materials with a density of 2.398 g cm^−3^ (see details in Tables S4 and S5).

In the Contact node, the Augmented Lagrangian method was employed to ensure an accurate contact pressure distribution, making it well-suited for modeling large deformations with plasticity. For the contact pairs, the upper side of the crystals was designated as the source boundaries (orange lines), while the lower side fibrils were defined as the destination boundaries (purple lines) (Fig. S32). Moreover, a prescribed displacement in the *y* direction (*dy*) of the crystal was set as 0.1 to 0.8 μm, with initial values set to zero. Before computation, the model was finely meshed using a stationary solver. To ensure accurate resolution of the contact area, the destination boundary of the contact pair was meshed finer than the source boundary by at least a factor of 2.

### Characterization

Rheological measurements were performed using an HR 20 rheometer (TA Instruments, USA) equipped with a 20.0-mm-diameter parallel plate, with the gap set to 1 mm. The CNF aqueous suspension was dispensed onto the bottom plate and allowed to rest for 5 min to minimize shear history caused by sample loading. Then, the gel was crosslinked with 1 ml of metal salt solution for 10 min before loading the top plate. To evaluate the viscoelastic properties, a frequency sweep was conducted at a constant strain of 0.1% within the linear viscoelastic region, with frequencies varying from 10^2^ to 10^−1^ rad s^−1^. Additionally, steady shear flow experiments were conducted across a shear rate range of 10^2^ to 10^−1^ s^−1^ to compare the apparent viscosity of different CNF aqueous suspensions. The tests were repeated 3 times to ensure reproducibility. Mechanical property was measured using a HY-0580 tensile tester (Shanghai Hengyi Precision Instrument Co., Ltd.) at a rate of 0.5 mm min^−1^. Raman spectra were recorded by a LabRAM HR Evolution system (Horiba France SAS) equipped with a 532-nm laser and an ND filter set to 10% transmission. The acquisition time was 20 s, with each spectrum obtained as the accumulation of 8 scans, and each measurement at the labeled area of the film was repeated 3 times for reproducibility. The spectra curves were fitted using the LabSpec 6 software to acquire the peak intensities and the hence the intensity ratio. Attenuated total reflection Fourier transform infrared (ATR-FTIR) was performed using an Alpha FTIR Spectrometer (Bruker, Germany). The spectra were recorded at room temperature in the range of 4,000 to 400 cm^−1^ as the average of 24 scans and a 4 cm^−1^ resolution. XPS measurements were carried out using a K-Alpha x-ray photoelectron spectrometer (Thermo Fisher Scientific, USA). A scanning electron microscope (Nova NanoSEM 450, FEI, USA) was used to characterize the sample morphologies under a voltage of 5 kV. XRD patterns were acquired using a Bruker D8 Discover diffractometer (Bruker AXS, Germany). Optical transmittance and haze were measured by a haze-gard I hazemeter (BYK-Gardner GmbH, Germany).

### Polarized UV–vis transmittance and theoretical calculation

A Filmetrics F20 spectrometer was utilized to measure the optical transmittance of the films at wavelengths of 400 to 800 nm. Thin film samples are placed between polarizers that are arranged in crossed or parallel configurations before measurement.

### Light retardation and birefringence

Transmission-mode POM measurements were conducted using a Nikon ECLIPSE E600 microscope. A Nikon LV100 microscope equipped with a Berek compensator was applied for measurement of light retardation, *Γ*, of the films. This compensator can be used as a variable waveplate that can impose a quarter- or half-wave retardation at any wavelength between 200 and 2,800 nm (5 wavelengths). Triple measurements in both clockwise and counterclockwise directions were performed to ensure accuracy (details presented in Note S3).

### Surface topography

The surface topography of the films was mapped by a Dektak 150 profilometer (Veeco Instruments Inc.). Measurements were performed at a speed of 0.27 mm/s and using constant pressure equivalent to a 5-mg tip weight to obtain the height profile. Note that the glass substrate exhibits a root mean square roughness of less than 1 nm. Therefore, the backside of the sample, adhered to the substrate, is considered flat, allowing the height data from the Dektak scan to be directly attributed to the film thickness.

## Data Availability

All data needed to evaluate the conclusions in the paper are present in the paper and/or the Supplementary Materials and are available from the corresponding authors upon request.

## Supplementary Materials

Supplementary 1 Notes S1 to S3 Figs. S1 to S32 Tables S1 to S5 Movies S1 to S3
