# Supplementary material for: Tunable Interference Colors in Nanofibril–Crystal Composite Films via Integrated Salt-Assisted Assembly
Source: Research (Wash D C). 2026 Mar 20;9:1198. doi: 10.34133/research.1198 (PMC13003156; doi:10.34133/research.1198)
Supplement: Supplementary 1 — Notes S1 to S3 Figs. S1 to S32 Tables S1 to S5 Movies S1 to S3 [file research.1198.f1.zip › 02_Supplementary Materials.docx]

Supplementary Materials

**Tunable Interference Colors in Nanofibril–Crystal Composite Films via Integrated Salt-Assisted Assembly**

Shaohuang Chen, Qun Song, Zengbin Wang, Yinqiang Xia, Philip Biehl, Rongxin Su*, and Kai Zhang*

**This file includes:**

Notes S1 to S3

Figures S1 to S32

Tables S1 to S5

Legends for movies S1 to S3

Supplementary References 1 to 23

**Other** **Supplementary Materials includes the following:**

Movies S1 to S3


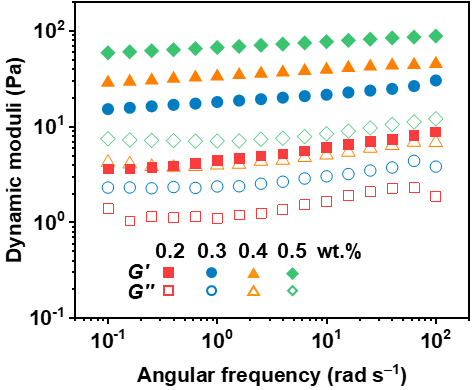


## Fig. S1 Dynamic moduli as a function of angular frequency for PCNF suspensions with concentrations of 0.2−0.5 wt.%.


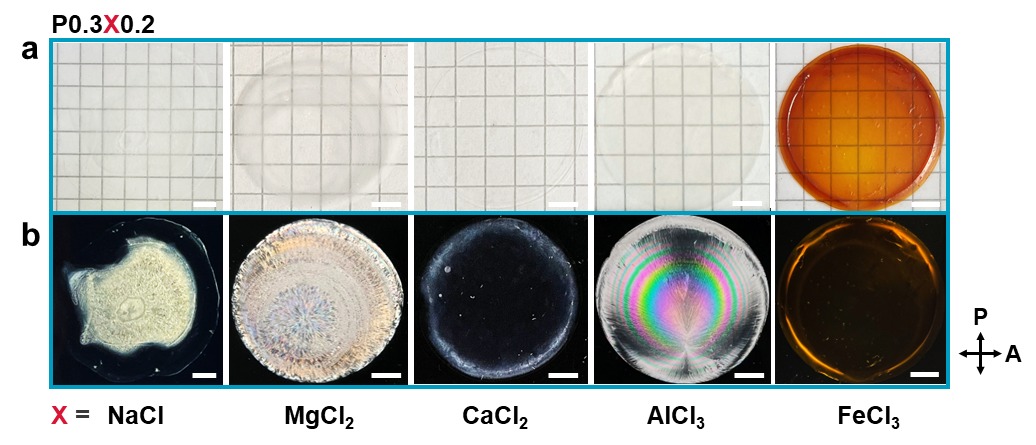


## Fig. S2 Effect of metal salt types on the film pattern. a) The films observed under natural light, showing high transparency. b) The films observed between crossed polarizers, showing varying surface patterns. P0.3 indicates 0.3 wt.% for PCNF suspensions and 0.2 indicates 0.2 mol L^–1^ for the metal chloride aqueous solutions. Crossed arrows indicate polarization axes of both linear polarizer and analyzer. Scale bars: 5 mm.


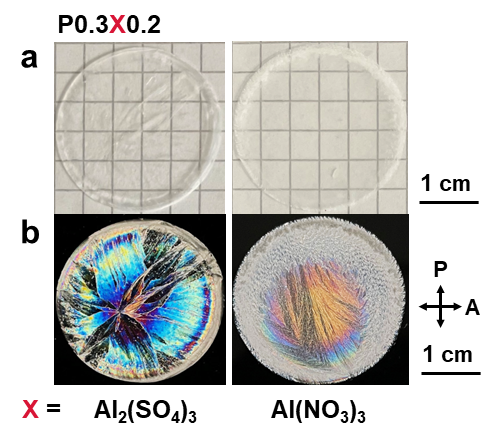


## Fig. S3 Effect of Al_2_(SO_4_)_3_ and Al(NO_3_)_3_ on the film pattern. a, b) The fabricated films observed under natural light (a) or polarized light (b). P0.3 indicates 0.3 wt.% for PCNF hydrogels and 0.2 indicates 0.2 mol L^–1^ for the aluminum salt aqueous solutions. The drying temperatures are 40 and 50 °C for the Al_2_(SO_4_)_3_ and Al(NO_3_)_3_ based samples, respectively.


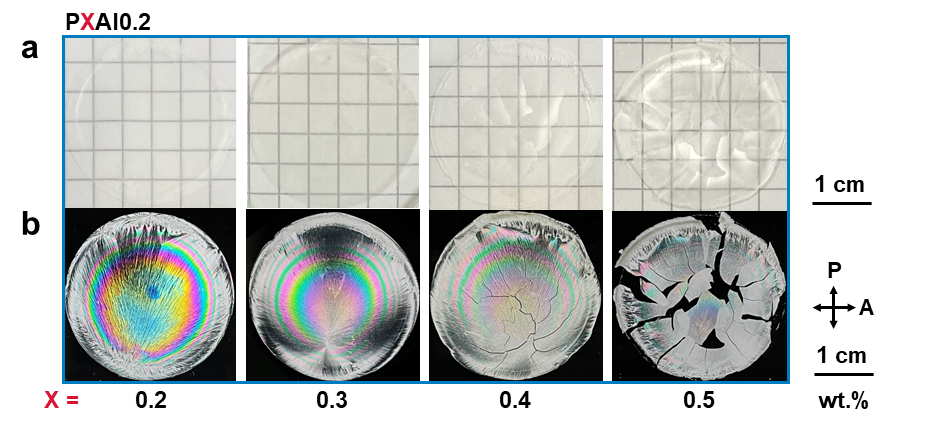


## Fig. S4 Effect of PCNF concentrations on the film pattern. a) Photographs of films prepared from PCNF suspensions crosslinked with 0.2 mol L^–1^ AlCl_3_ solutions (PXAl0.2). X indicates PCNF concentrations (0.2–0.5 wt.%). b) The corresponding films observed under crossed polarizers.


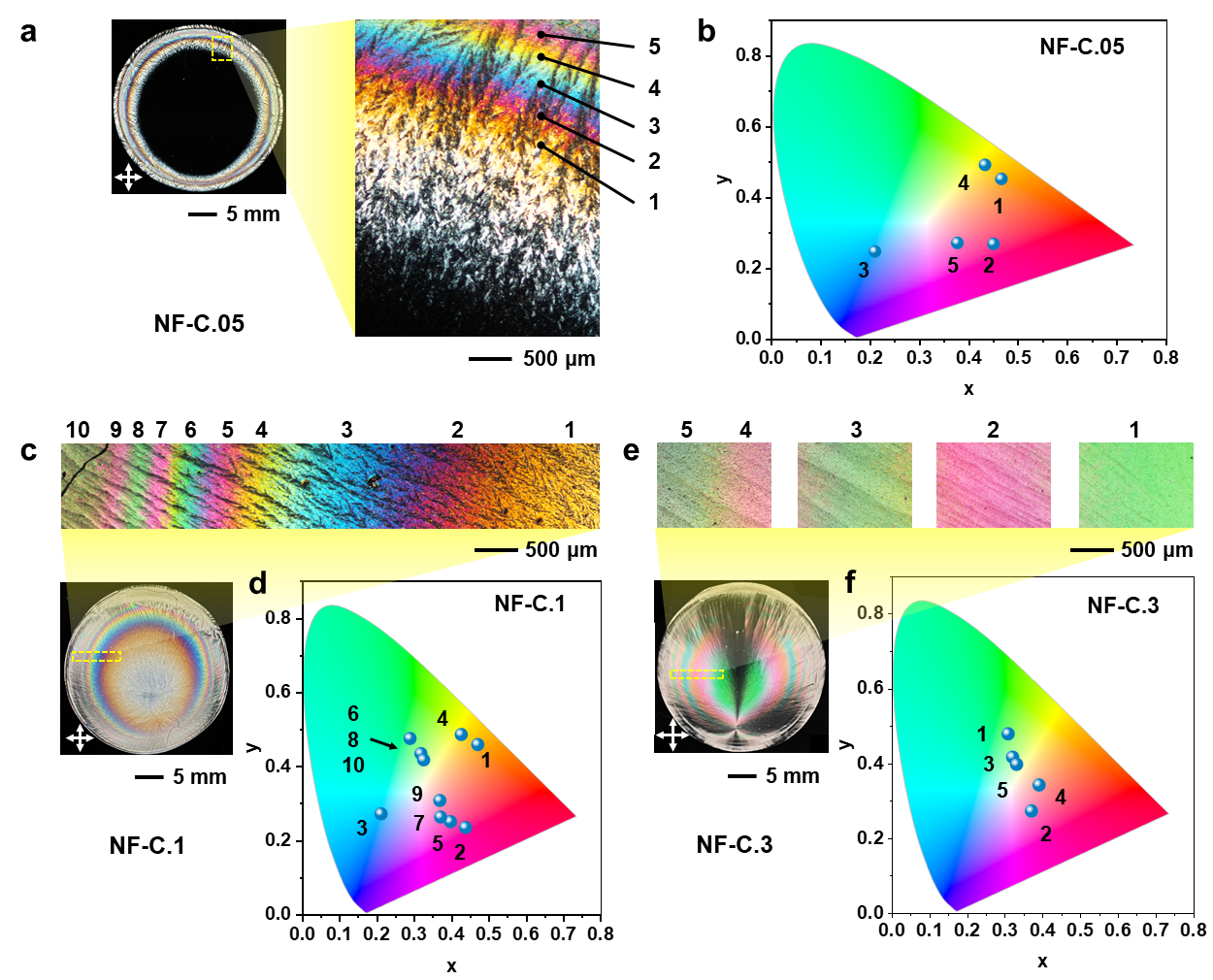


## Fig. S5 POM images (a, c, e) and chromaticity coordinates on CIE diagram (b, d, f) of the different positions of the NF-C complex films prepared using different concentrations of AlCl_3_.


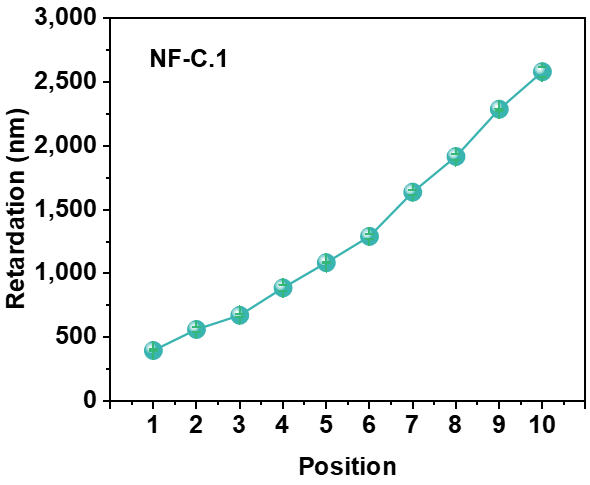


## Fig. S6 Light retardation of various positions (1–10) of the P0.3Al0.1 film. Error bars represent standard deviations calculated from three independent measurements (*n* = 3).


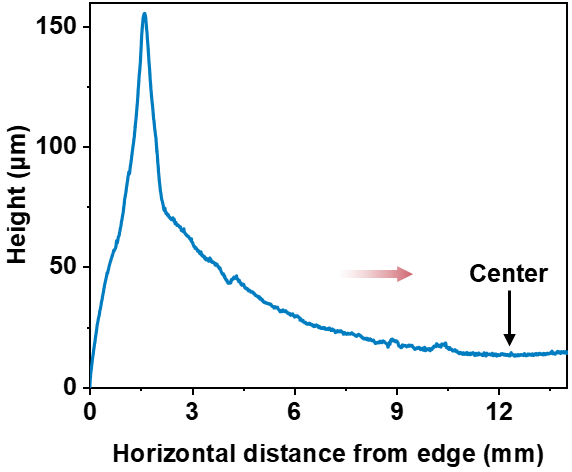


## Fig. S7 Height profile of NF-C.2 obtained from a profilometer.


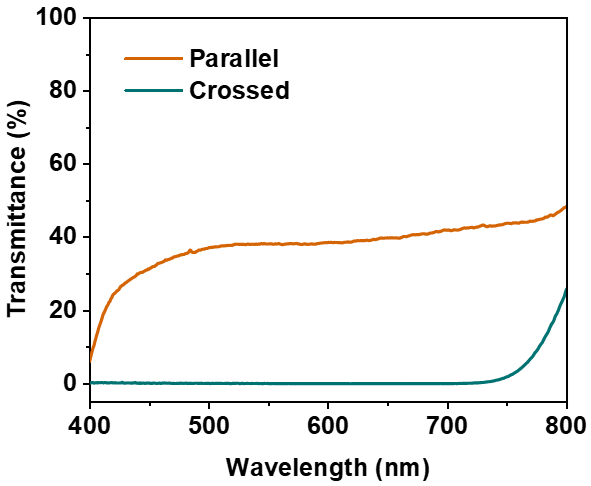


## Fig. S8 UV-vis spectra of crossed and parallel polarizers.


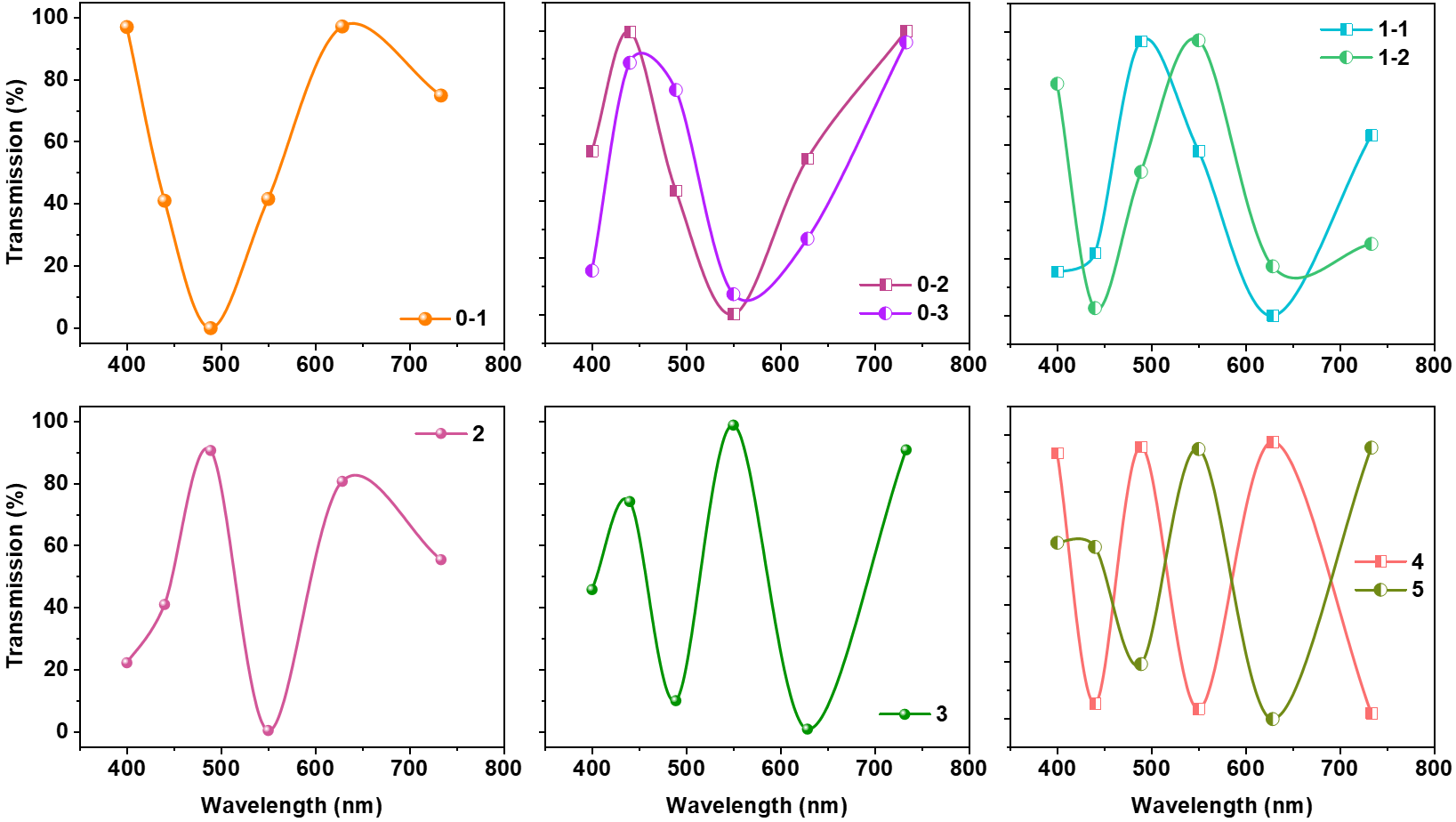


## Fig. S9 Theoretical calculation of the transmission intensity at varying positions of the NF-C.2 film. The analyzer is polarized 90° to the polarizer (crossed arrangement).


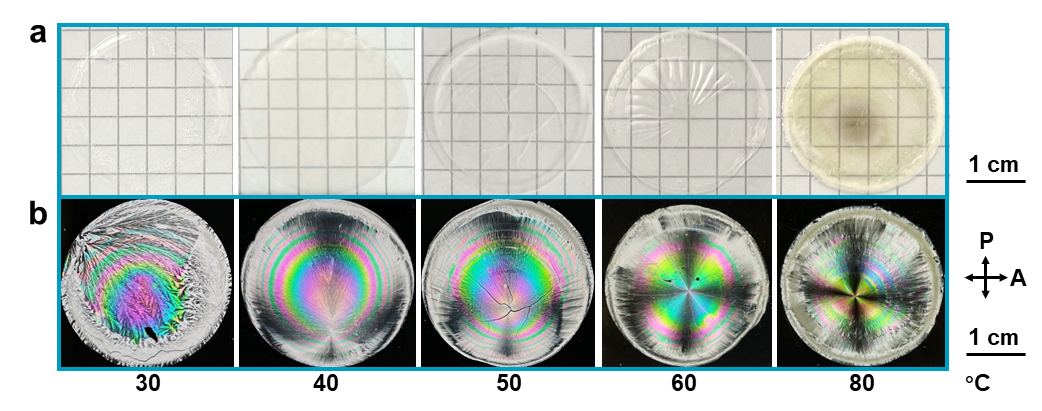


## Fig. S10 Effect of drying temperatures on the surface pattern of P0.3Al0.2 films. a, b) Photographs of the samples prepared at temperatures ranging from 30 to 80 °C (a), and the corresponding ones observed between crossed polarizers (b).


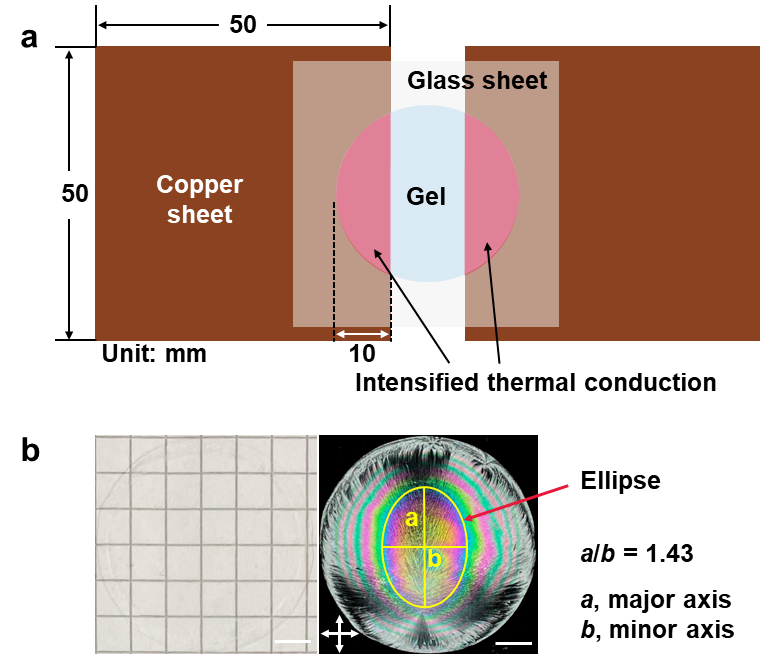


## Fig. S11 Tailoring of surface pattern by uneven heat transfer. a) Scheme of the experimental set-up, including crosslinked hydrogel, glass substrate, and two copper sheets. Two copper sheet is introduced to induce uneven heat distribution within the gel during evaporation. b) Photograph of the resulting film observed under natural light (left) and polarized light (right). Scale bars: 5 mm.


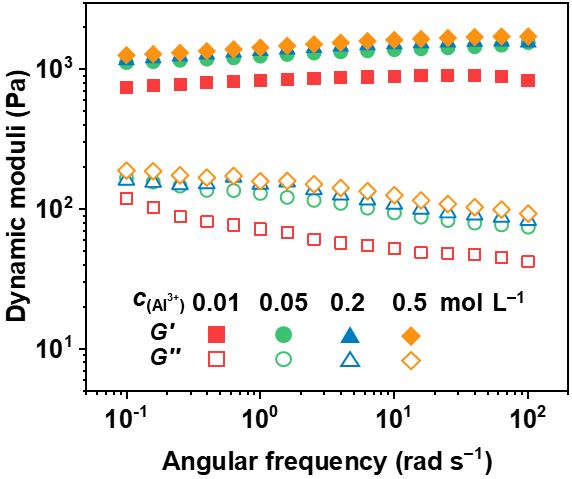


## Fig. S12 Dynamic moduli as a function of angular frequency for PCNF suspension (0.3 wt.%) crosslinked with various concentrations of AlCl_3_ solutions (0.01–0.5 mol L^–1^).


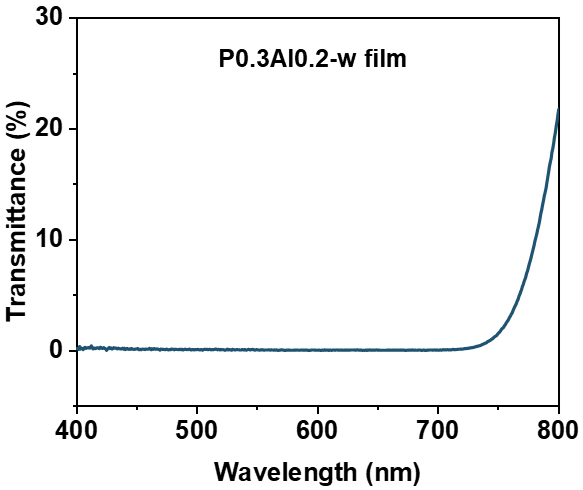


## Fig. S13 Polarized UV-vis transmission spectra of the P0.3Al0.2-w film under crossed polarizers.

## Note S1: Raman spectra of P0.3Al0.2-w

As shown in **Fig. S14**, the characteristic bands at 200−600 cm^−1^ correspond to the vibrational modes of AlO_6_ in crystalline AlCl_3_·6H_2_O [1]. Specifically, the low-frequency bands (< 350 cm^−1^) are primarily originated from internal vibrations of the AlO_6_ group, with peaks at 293 and 306 cm^−1^ assigned to *A*_1g_ and *E*_g_ modes, respectively. In the Raman spectra of the P0.3Al0.2-w film, the characteristic bands of AlCl_3_·6H_2_O disappear, and new vibration bonds emerge at 348, 379, 437, and 459 cm^−1^ (**Fig. S15**), corresponding to the bending motions of cellulose molecule [2,3]. The predominant motions of cellulose within the low-energy vibrational region (250−550 cm^−1^) involve skeletal-bending modes associated with the CCC, COC, OCC, and OCO internal coordinates [3].


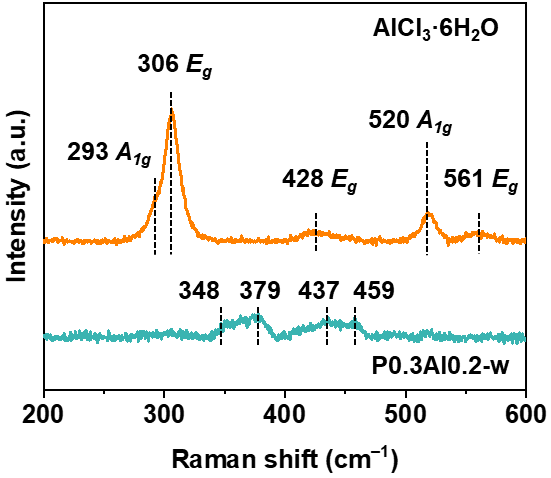


## Fig. S14 Raman spectra of AlCl_3_·6H_2_O compound and P0.3Al0.2-w.


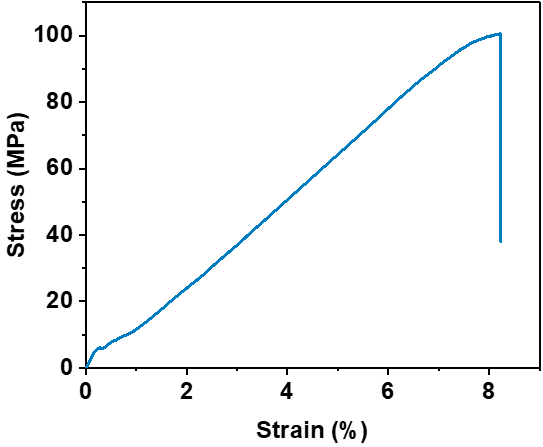


## Fig. S15 Tensile stress–strain curve of the films prepared from the gels after washing.

**
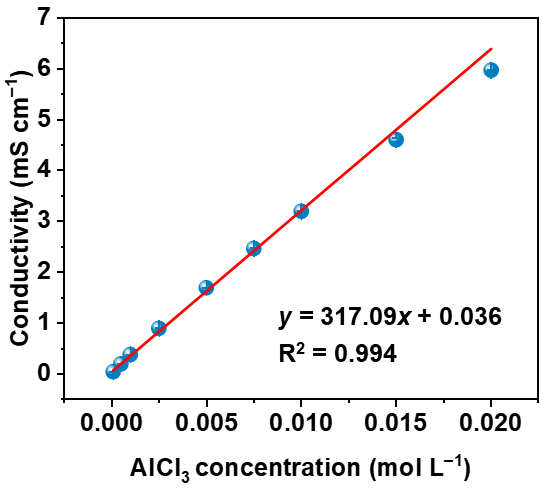
**

## **Fig. S16** Conductivity of AlCl_3_ aqueous solution as a function of concentration (0.0001–0.02 mol L^–1^). Test temperature: 25 ℃.


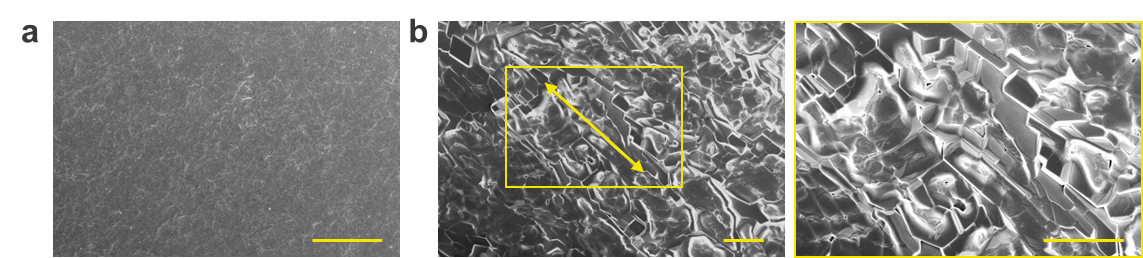


## Fig. S17 a, b) SEM image of surface of the P0.2Al0.2-w (a) and NF-C.2 film (b). The double-headed arrows in (b) indicate the orientation of the crystals. Scale bars: 5 μm.


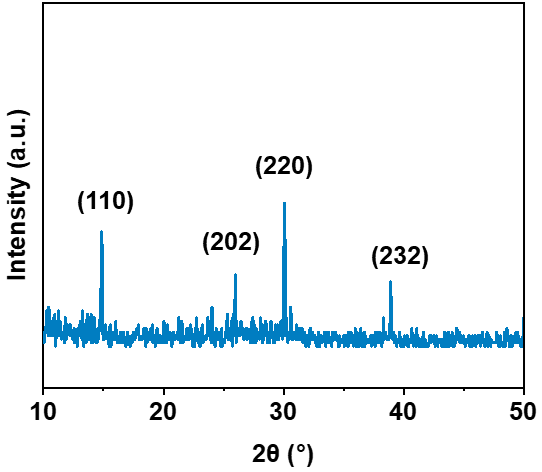


## Fig. S18 XRD spectra of the NF-C.2 film, indicating the presence of AlCl_3_·6H_2_O crystals.


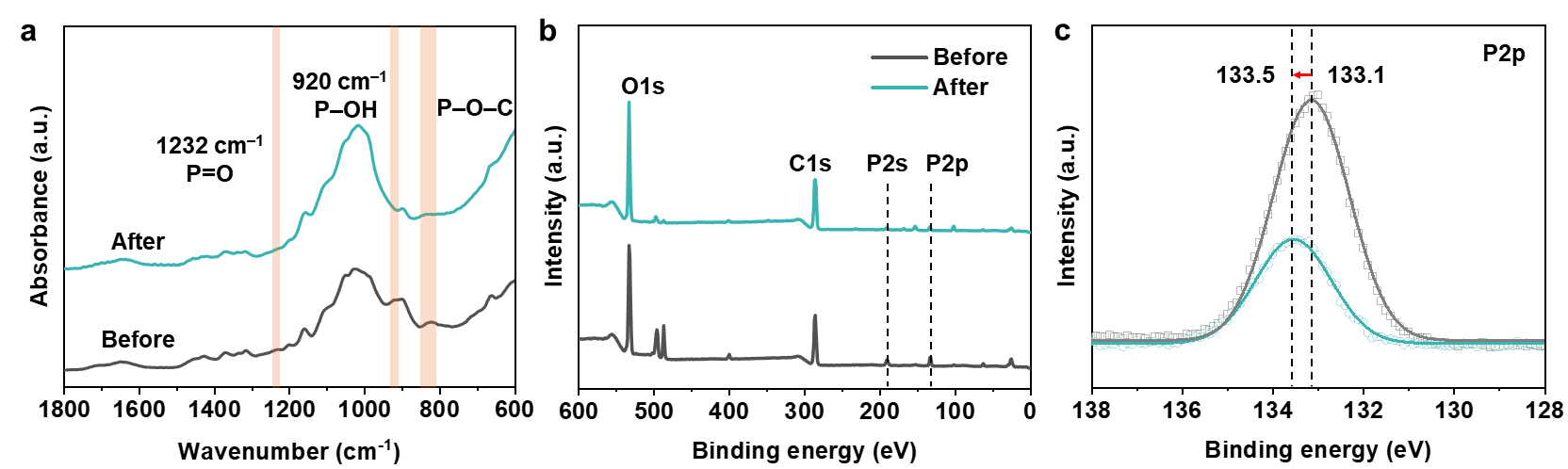


## Fig. S19 Characterization of the PCNFs film before and after crosslinking with 0.2 M AlCl_3_.

a) ATR-FTIR spectra. b, c) XPS spectra (b) and high-resolution scans of P 2p analysis (c). Note: P0.3Al0.2-w is used to represent the crosslinked film for the chemical bonding analysis.


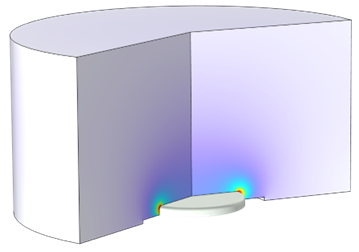


## Fig. S20 Numerical simulations of 3D moisture flux distribution over the gel surface after evaporation at 40 ℃ for 60 s.


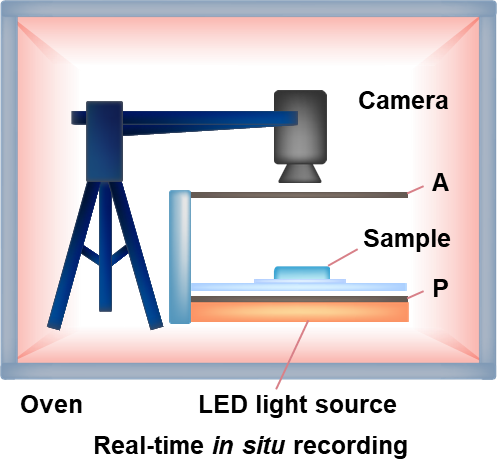


## Fig. S21 Schematic illustration of the experimental setup used to monitor the drying process of the crosslinked hydrogel. The camera is connected to a laptop, which is not presented in the scheme. P, polarizer; A, analyzer.


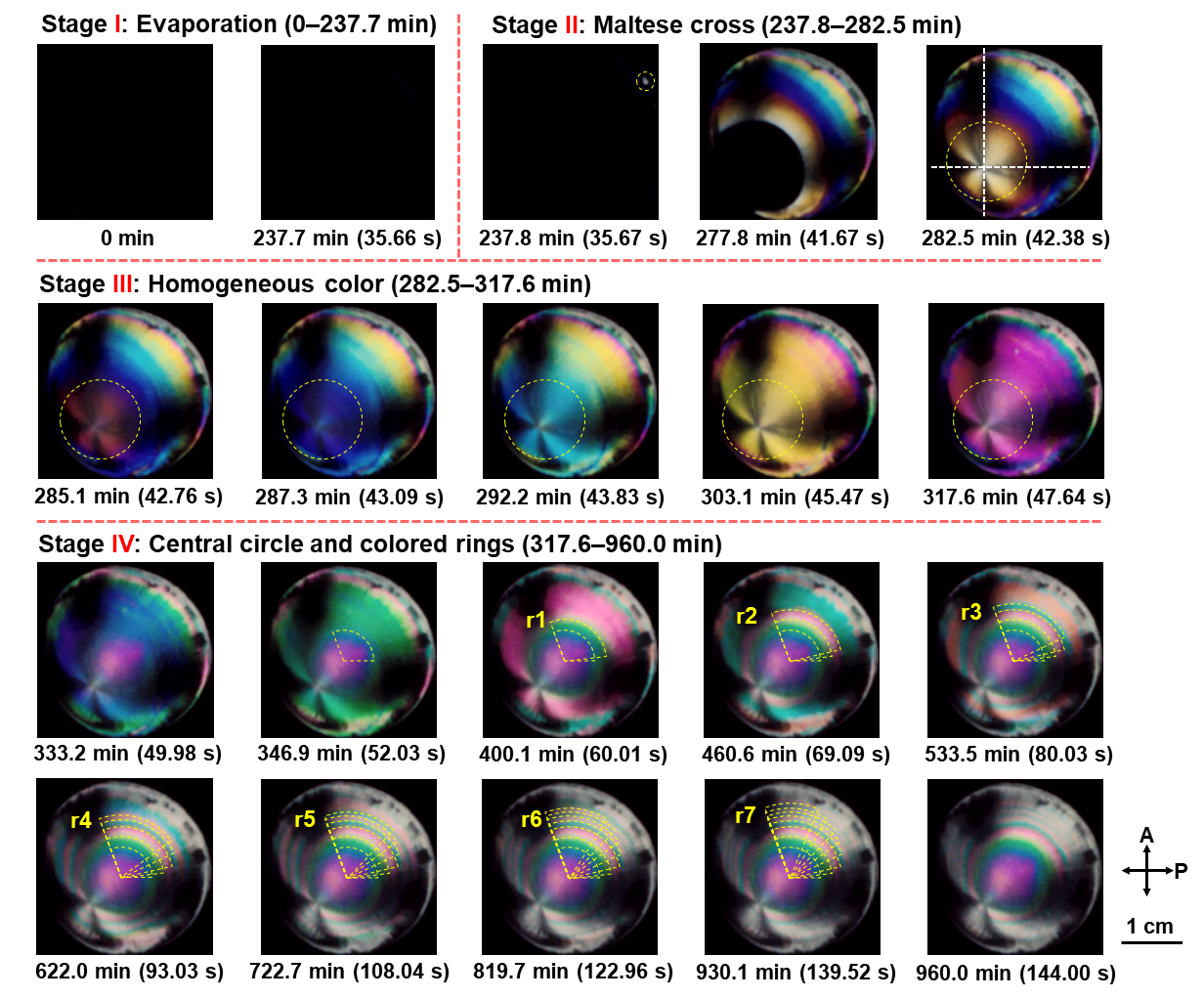


## Fig. S22 Evolution of the chromatic pattern of the NF-C.2 film. The snapshots of the film taken at different drying times, showing the evolution of the spatiotemporal pattern. The process can be divided into four stages, including water evaporation of the crosslinked hydrogel (stage I), formation of characteristic Maltese cross (stage II), homogeneous colors in the labeled circular area (stage III), and central circle and colored rings (stage IV). r1–7 indicate the seven colored rings. The numbers in parenthesis with unit second correspond to the times in the accelerated Movie (Movie S1).


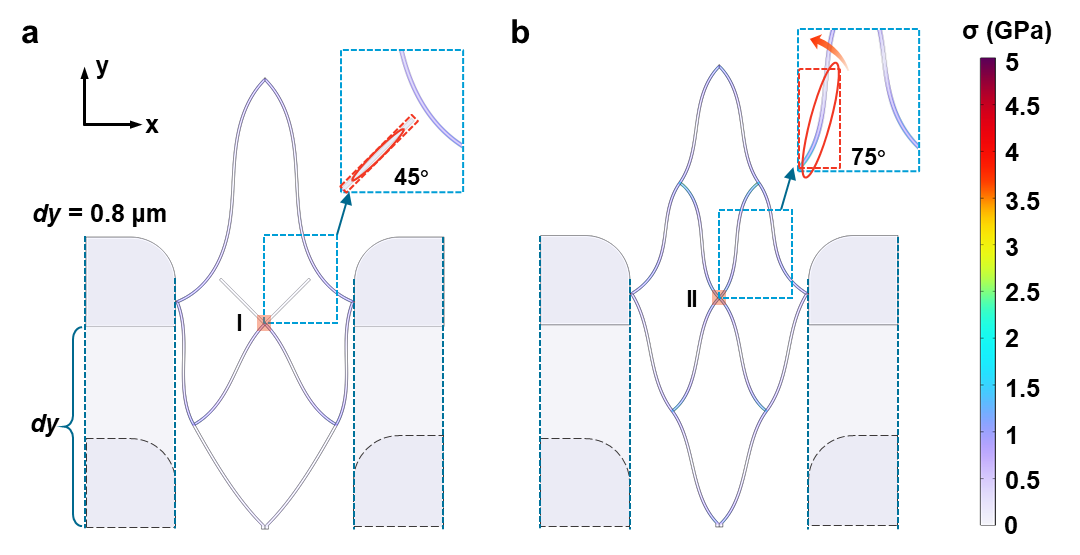


## Fig. S23 Deformation of nanofibril network upon contact with crystals. a, b) Deformation of the nanofibril networks with the internal fibrils disconnected (a, control) from or connected (b, NF-C network) to the upper adjacent ones under a 0.8-μm displacement in the *y* direction (*dy*) of the crystals. Zoom-in images of the area labeled with blue dash line indicate the deformation of the internal fibrils upon contact with the crystals.


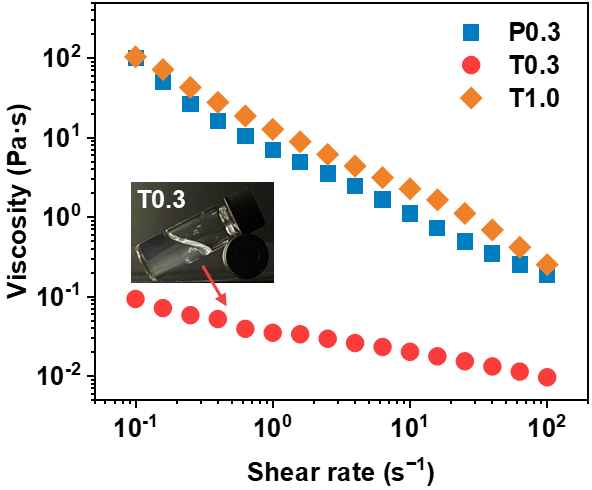


## Fig. S24 Viscosity of CNF suspensions as functions of shear rate and concentration. P0.3, T0.3, and T1.0 indicate 0.3 wt.% PCNF, 0.3 wt.% TOCNF, and 1.0 wt.% TOCNF suspensions, respectively. The inset is a photograph of T0.3, showing liquid-like behaviors.


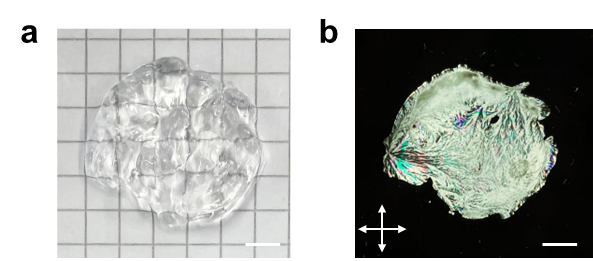


## Fig. S25 a, b) Photograph of T0.3 crosslinked with 0.2 M AlCl_3_ (a) and the resulting polarization pattern (b). Scale bars: 5 mm.


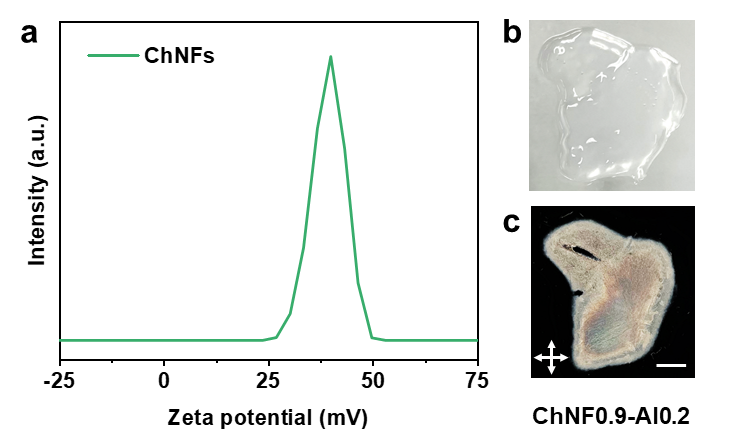


## Fig. S26 a) Zeta potential distribution of 0.1 wt. % chitin nanofibril (ChNF) suspensions. b, c) ChNF hydrogel (0.9 wt.%) crosslinked with 0.2 M AlCl_3_ (ChNF0.9-Al0.2, b) and the resulting film observed between cross polarizers (c). Scale bar: 5 mm.


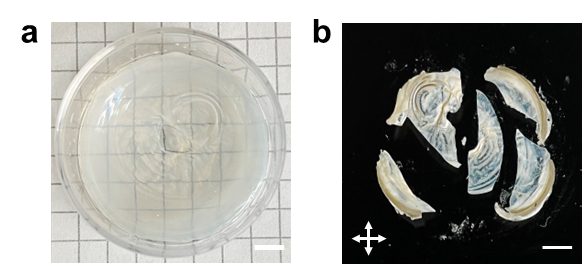


## Fig. S27 a) Cellulose phosphate (CP) solution (10 wt.%) crosslinked with 0.2 M AlCl_3_ (CP10Al0.2). b) The CP10Al0.2 film observed between cross polarizers. Note: the gel cracked when it was transferred onto the glass substrate before drying in an oven. Scale bars: 5 mm.


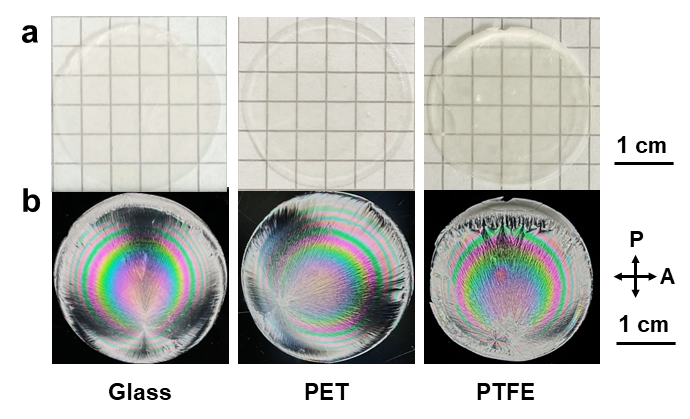


**Fig. S28** Demonstration of applicability on representative substrates including glass, polyethylene terephthalate (PET), and polytetrafluoroethylene (PTFE). a, b) Photographs of P0.3Al0.2 (i.e., NF-C.2) films under natural light (a) and polarized light (b).


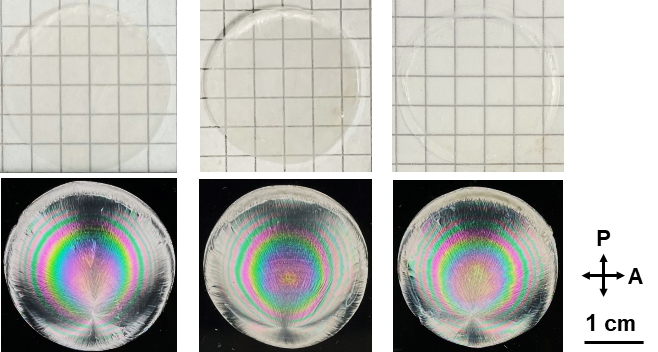


## Fig. S29 Demonstration of reproducibility of the NF-C.2 film.

**
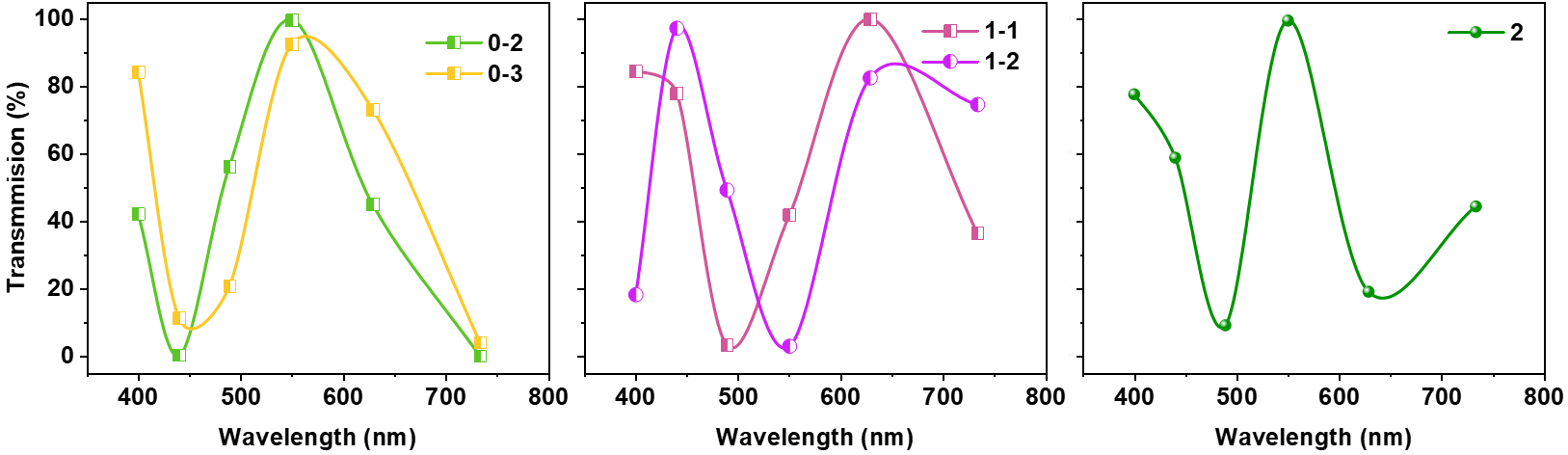
**

## Fig. S30 Theoretical calculation of the transmission intensity at varying positions of the NF-C.2 film. The analyzer is polarized 0° to the polarizer (parallel arrangement).

## Note S2: Numerical simulation of hydrogel evaporation

Evaporation is a process where molecules at the liquid surface gain energy to transition into the vapor phase, which is driven by the difference in vapor concentration between the liquid surface and the surrounding air (concentration gradient). This equilibrium process is obtained until the air reaches its saturated vapor concentration (*c*_sat_) at a given temperature.

For ideal gases, the saturation concentration is determined with:

$c_{sat}\left( T \right)=\frac{p_{sat}(T)}{RT}$ (1)

where *R* is the ideal gas constant and *p*_sat_ is the saturation pressure.

The *p*_sat_ is strongly temperature (*T*) dependent and can be defined by the following approximation [4,5]:

$p_{sat}(T)=611\exp^{17.27\frac{T-273.15}{T-35.85}}$ (2)

E**vaporation** at a surface results in a transfer of mass (moisture) between the surface and the surrounding air, creating a **net moisture flux** (**J_m_**​) at the surface. Considering steady state, this net moisture flux corresponds to an effective convective velocity at the domain boundary, i.e., the Stefan velocity (𝒖_s_) [6], which is defined by:

$\rho_{g}u_{s}= \mathbf{J}_{\mathbf{m}}$ (3)

where *ρ*_g_​ is the density of the gas phase, kg m^−3^. For a mixture of dry air and water vapor, *ρ*_g_ includes both components.

In COMSOL Multiphysics, the moisture flux (**J_m_**) is computed as follows:

$\mathbf{J}_{\mathbf{m}}\boldsymbol{= -}M_{v}D▽c_{v}$ (4)

$c_{v}=\Phi c_{sat}$ (5)

where *M*_v_ is the molar mass of vapor, g mol^−1^, *D* is the diffusion coefficient, and *Φ* is the relative humidity.

Additionally, the wet surface feature is assigned to the gel surface to implement the source term for the water vapor, as defined by the following equations:

${-\mathbf{J}_{\mathbf{m}}=J}_{evap}$ (6)

$J_{evap}=M_{v}K\left( c_{sat}-c_{v} \right)$ (7)

where *K* is the evaporation rate factor.

The initial temperature (*T*_0_) and relative humidity (*Φ*_0_) are set as 40 °C and 30%, respectively, to simulate the surroundings in the oven.

## Note S3: Determination of light retardation

The Berek Compensator, invented by German mineralogist Max Berek in 1913, is primarily used as an accessory for a polarized light microscope, allowing for quantitative analysis of optical retardation [7]. This type of polarization compensator can be used as a variable waveplate that can impose a quarter or half-wave retardation at any wavelength between 200 and 2,800 nanometers (5 wavelengths).

The measurement was performed by tilting the compensator plate until an extinction of the light transmitted by the sample is detected, as outlined in the Berek Compensator instructions. Briefly, the sample was rotated until its optic axis was positioned at 45° to the polarizers, maximizing the transmitted light. Next, the slow directions of the sample and the Berek compensator were aligned perpendicularly to each other to achieve the subtraction position. Then, the barrel of the compensator was rotated until the black band appears at the center of the viewfield, indicating the extinction position. Because the plate can tilt on either side of the optical axis, taking one measurement on each side and averaging the two values enhances accuracy. The readings for the clockwise and counterclockwise directions were recorded as *a* and *b*, respectively, and the inclination degree *i* was calculated as follows:

$i=\frac{a-b}{2}$ (8)

The retardation *Γ* can be obtained from *i* using the following equation:

$\Gamma= f(i) C_{\lambda}$ (9)

$f(i) = \sin^{2} i(1 + 0.2040 \sin^{2} i + 0.0627 \sin^{4} i)$ (10)

where *f*(*i*) is a tabulated function and *C_λ_* is the machine constant.

When conducting measurements under white light, the machine constant *C_λ_* provided by the manufacturers is 10050, corresponding to *λ* = 550 nm, which is at the center of the spectrum.


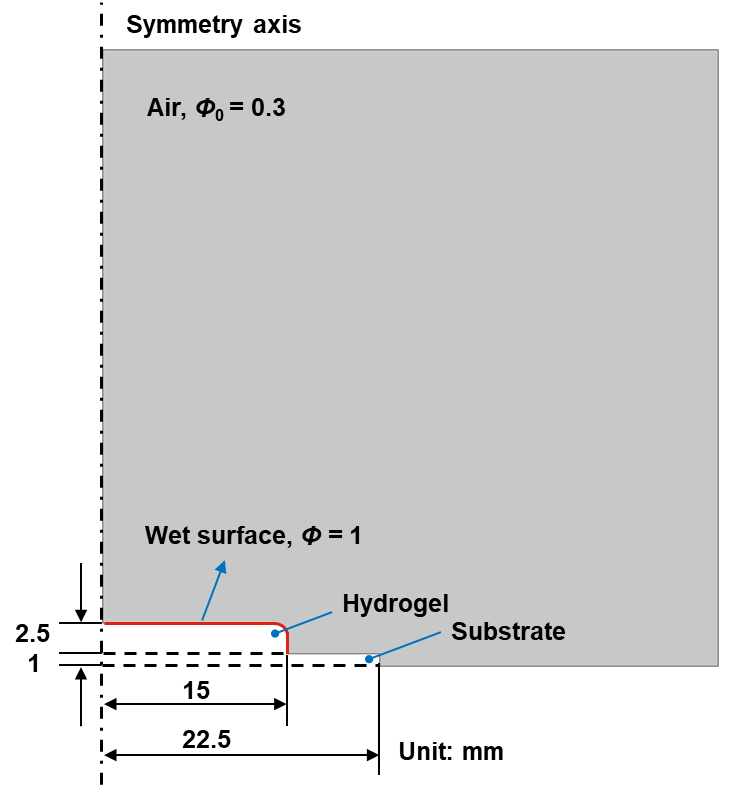


## Fig. S31 The 2D axisymmetric geometry of the hydrogel evaporation in air. The fillet radius was 1 mm.


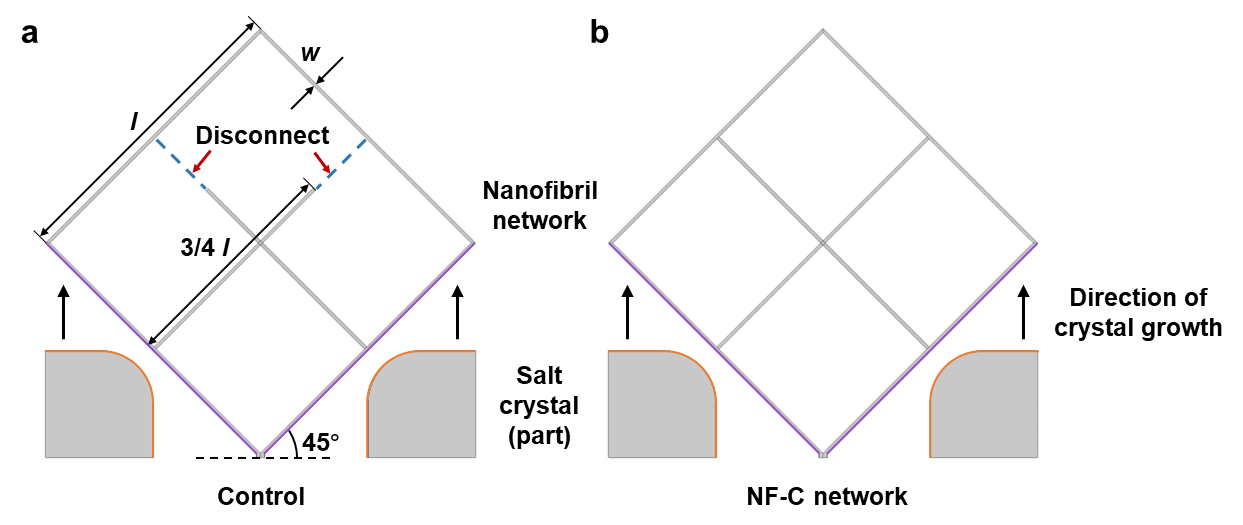


## Fig. S32 Construction of COMSOL models. a, b) Model geometry of crystals and nanofibril networks with the internal fibrils disconnected (a) from or connected (b) to the upper adjacent ones, which are used as a control group and to simulate the NF-C network, respectively. Note: the orange lines of the crystals are the source boundaries, while the purple lines the fibrils indicate the destination boundaries.

## Table S1. Comparison of fabrication method, size, and application of nanocellulose-based optical materials exhibiting interference colors.

| **Material** | **Fabrication method** | **Area *^a^***  **(or width *^b^*)** | **Application** | **Ref** |
| --- | --- | --- | --- | --- |
| **NF-C** | **iSAA** | **680** | **Spectrally selective polychromatic lighting** | **This work** |
| L-(+)-TA/  CNC | EISA; reaction–diffusion | 4.9 *^c^* | Design of self-organizing, periodically structured materials | [8] |
| CNC/NL | EISA | 113 | Matrix information encryption | [9] |
| PO-CNC/GNR | Stretching | 1.9 *^c^* | Tuning of multiple interference colors | [10] |
| CNC/GNR | Stretching | 110 | Information encryption and photonic displays | [11] |
| PAM/CNC | Stretching | Width < 1.3 *^c^* | Optical coding-based information encryption | [12] |
| CNC-dynamic hydrogel | Stretching | Width < 2.5 *^c^* | N/A | [13] |
| CNC-elastomer | Stretching | Width < 7.1 | Stimuli-responsive flexible optics | [14] |
| CNC elastomer | Stretching | Width < 10 | N/A | [15] |
| CNC/PVA | Stretching | Width < 5 | Information encryption and identification | [16] |

*^a^* unit: mm^2^; *^b^* unit: mm; *^c^* observed by polarized optical microscope (POM)

NF-C, nanofibril-crystal compositeL-(+)-TA, L-(+)-tartaric acid; NL, nanolignin; PO-CNC, periodate-oxidized CNC; GNR, gold nanorod; PAM, polyacrylamide; PVA, polyvinyl alcohol; iSAA, integrated salt-assisted assembly; EISA, evaporation-induced self-assembly; N/A, not available.

## Table S2. Material consumption per square meter of NF-C.2 film produced based on the lab-scale data.

| **Material** | **Quantity (lab use)** | **Unit** | **Quantity** | **Unit** |
| --- | --- | --- | --- | --- |
| PCNFs | 0.0075 | g | 0.0106 | kg |
| AlCl_3_·6H_2_O | 0.2414 | g | 0.3416 | kg |
| NF-C.2 film | 7.0686 | cm^2^ | 1 | m^2^ |

## Table S3. Estimated material costs per square meter of NF-C.2 film produced.

| **Material** | **Usage (kg)** | **Industrial price ($/ton)** | **Costs ($)** | **Source of price** |
| --- | --- | --- | --- | --- |
| PCNFs | 0.0106 | 1080 | 0.01 | Ref.[17] |
| Aluminium chloride hexahydrate | 0.3416 | 330 | 0.11 | https://www.alibaba.com/product-detail/Industrial-water-treatment-Sell-Well-New_1600603880364.html?spm=a2700.7724857.0.0.53f4549fv2uUJ5 |
| Total | / | / | 0.12 | **/** |

## Table S4. Physical properties of nanocellulose.

| **Material** | **Size (nm)** | **Density**  **(g cm^−3^)** | **Elastic modulus**  **(GPa)** | **Poisson ratio** | **Ref.** |
| --- | --- | --- | --- | --- | --- |
| Cellulose I_β_ | / | 1.6 | 120–138 | 0.38 | [18-20] |
| Single CNFs*^a^* | *d*: 35−90 | / | 78±17 | / | [21] |
| Single CNC*^b^* | *d:*10  *l:* 100–150 | / | 57−105 | / | [22] |
| Macro-fibers from CNFs*^c^* (orientation index: 0.92) | *d:* 6.8 ± 0.9 μm | / | ∼70 | / | [23] |

*^a^* sourced *from* bacterial. *^b^* sourced from cotton. *^c^* sourced from chemically bleached wood fibers.

*l*: length. *d*, diameter.

## Table S5. Material properties used in the numerical simulation.

| **Material** | **Size** | **Density (g cm^−3^)** | **Elastic modulus (GPa)** | **Poisson ratio** | **Ref.** |
| --- | --- | --- | --- | --- | --- |
| CNFs | *w*: 10 nm  *l*: 1 μm | 1.6 | 80 | 0.38 | Table S4 |
| AlCl_3_·6H_2_O | / | 2.398 | / | / | / |

**Legends for Movies S1–S3**

**File Name:** Movie S1

**Description:** Drying process of the crosslinked hydrogel, exhibiting an order-to-disorder transition of the polarization pattern.

**File Name:** Movie S2

**Description:** Polychromatic lighting observed under a horizontal view.

**File Name:** Movie S3

**Description:** Polychromatic lighting observed under a top view.

## Supplementary References

1. Adams D M, Hills D J, Single-crystal raman and infrared study of aluminium trichloride hexa-hydrate. *Journal of the Chemical Society, Dalton Transactions.* 1978;7:782–788.

2. Agarwal U P, "Raman spectroscopy of cnc- and cnf-based nanocomposites" in *Handbook of nanocellulose and cellulose nanocomposites* (Wiley-VCH, Weinheim, Germany, 2017), pp. 609–625.

3. Wiley J H, Atalla R H, Band assignments in the raman spectra of celluloses. *Carbohydr. Res.* 1987;160:113–129.

4. Monteith J L, Unsworth M H, Webb A, Principles of environmental physics. *Q. J. R. Meteorol. Soc.* 1994;120(520):1699.

5. Murray F W, On the computation of saturation vapor pressure. *J. Appl. Meteorol. Climatol.* 1967;6(1):203–204.

6. Xu D, Meng X, Liu S, Poisson J, Vana P, Zhang K, Dehydration regulates structural reorganization of dynamic hydrogels. *Nat. Commun.* 2024;15(1):6886.

7. Durey G, Polarized microscopy with the berek compensator: A comprehensive tutorial for the modern reader. *Eur. Phys. J. Plus.* 2021;136(8):866.

8. Ackroyd A J, Holló G, Mundoor H, Zhang H, Gang O, Smalyukh I I, Lagzi I, Kumacheva E, Self-organization of nanoparticles and molecules in periodic liesegang-type structures. *Sci. Adv.* 2021;7(16):eabe3801.

9. Song Q, Meng X, Huang C, Xu M, Chen T, Zhou Z, Vana P, Zhang K, Self-assembled heterosymmetric structure with tunable polarization optics for reversible matrix encryption. *Adv. Funct. Mater.* 2025;35(46):2511897.

10. Xu D, Song Q, Wu C, Zhang K, Designable multiple structural colors using alkaline periodate oxidated cellulose nanocrystals and gold nanorods. *Adv. Mater. Technol.* 2022;7(12):2200615.

11. Wang X, Xu D, Jaquet B, Yang Y, Wang J, Huang H, Chen Y, Gerhard C, Zhang K, Structural colors by synergistic birefringence and surface plasmon resonance. *ACS Nano.* 2020;14(12):16832–16839.

12. Yang Y, Wang X, Huang H, Cui S, Chen Y, Wang X, Zhang K, Modular nanocomposite films with tunable physical organization of cellulose nanocrystals for photonic encryption. *Adv. Opt. Mater.* 2020;8(12):2000547.

13. Huang H, Wang X, Yu J, Chen Y, Ji H, Zhang Y, Rehfeldt F, Wang Y, Zhang K, Liquid-behaviors-assisted fabrication of multidimensional birefringent materials from dynamic hybrid hydrogels. *ACS Nano.* 2019;13(4):3867–3874.

14. Kose O, Tran A, Lewis L, Hamad W Y, MacLachlan M J, Unwinding a spiral of cellulose nanocrystals for stimuli-responsive stretchable optics. *Nat. Commun.* 2019;10(1):510.

15. Kose O, Boott C E, Hamad W Y, MacLachlan M J, Stimuli-responsive anisotropic materials based on unidirectional organization of cellulose nanocrystals in an elastomer. *Macromolecules.* 2019;52(14):5317-5324.

16. Sun W, Wang J, He M, Anisotropic cellulose nanocrystal composite hydrogel for multiple responses and information encryption. *Carbohydr. Polym.* 2023;303:120446.

17. Chen S, Xu D, Yin H, Huang R, Qi W, Su R, Zhang K, Large-scale engineerable films tailored with cellulose nanofibrils for lighting management and thermal insulation. *Small.* 2024;20(43):2401283.

18. Moon R J, Martini A, Nairn J, Simonsen J, Youngblood J, Cellulose nanomaterials review: Structure, properties and nanocomposites. *Chem. Soc. Rev.* 2011;40(7):3941–3994.

19. Nakamura K I, Wada M, Kuga S, Okano T, Poisson's ratio of cellulose iβ and cellulose ii. *J. Polym. Sci. Part B: Polym. Phys.* 2004;42(7):1206–1211.

20. Tavares da Costa M V, Berglund L A, Modeling of modulus and strength in void-containing clay platelet/cellulose nanocomposites by unit cell approach. *Nanocomposites.* 2023;9(1):138–147.

21. Guhados G, Wan W, Hutter J L, Measurement of the elastic modulus of single bacterial cellulose fibers using atomic force microscopy. *Langmuir.* 2005;21(14):6642–6646.

22. Rusli R, Eichhorn S J, Determination of the stiffness of cellulose nanowhiskers and the fiber-matrix interface in a nanocomposite using raman spectroscopy. *Appl. Phys. Lett.* 2008;93(3):033111.

23. Mittal N, Ansari F, Gowda.V K, Brouzet C, Chen P, Larsson P T, Roth S V, Lundell F, Wågberg L, Kotov N A *et al.*, Multiscale control of nanocellulose assembly: Transferring remarkable nanoscale fibril mechanics to macroscale fibers. *ACS Nano.* 2018;12(7):6378–6388.
